# M^6^A-METTL3-dependent nuclear PANC754/PSPC1/H3K4me1 repression complex regulate immune evasive LGALS7 signal to enhance immunotherapy against colorectal cancer

**DOI:** 10.1038/s41419-025-07820-9

**Published:** 2025-07-09

**Authors:** Jianfeng Zhang, Guilian Cao, Feng Li, Siyu Tang, Chenxi Wu, Min Jiang, Jingxin Ye, Shaoqing Ju, Fei Qian, Weifeng Ding

**Affiliations:** 1 Department of Laboratory Medicine; Department of Gastroenterology, Affiliated Hospital of Nantong University, Medical School of Nantong University, Nantong, Jiangsu Province China; 2 Department of Laboratory Medicine, The Third People’s Hospital of Changzhou, Changzhou, Jiangsu Province China; 3 Laboratory Medicine Center, The Sixth People’s Hospital of Nantong (Affiliated Nantong Hospital of Shanghai University), Nantong, Jiangsu Province China; 4 Department of Gastroenterology, The Affiliated Suqian Hospital of Xuzhou Medical University, Suqian, Jiangsu Province China; 5 Department of Gastrointestinal Surgery, Affiliated Hospital of Nantong University, Medical School of Nantong University, Nantong, Jiangsu Province China

**Keywords:** Genetics research, Colorectal cancer

## Abstract

Non-coding RNAs (ncRNAs) have important regulatory functions similar to traditional oncogenes or tumor suppressor genes. Our previous research found a novel pan-cancer downexpressed ncRNA, *PANC754*. However, its function and underlying mechanism remain obscure in colorectal cancer (CRC). In this study, in vitro and in vivo experiments were performed to determine the function of *PANC754*. Loss and gain of function experiments, molecular docking experiments, and bioinformatic analysis were utilized to visualize its pathway. Co-culture system was leveraged to explore its effect on synergetic immune checkpoint blockage against CRC. Through a series of studies, we found that overexpressed *PANC754* significantly inhibited cell viability, migration, and metastasis and induced notable apoptosis in CRC. The mechanical research found that PANC754 was the nuclear-located and its expression was regulated by m^6^A modification via METTL3 enzyme, which bound with its RBP PSPC1, then interacted with H3K4me1 to chromatin-accessible inhibit immune evasive molecule LGALS7 and led to suppress CRC progress. Furthermore, we confirmed that prominent upregulation of the immune checkpoint inhibitory (ICI) capability of anti-NKG2A, monalizumab when it was combined with PANC754 overexpression. Collectively, our study revealed that *PANC754* is a tumor-suppressing ncRNA to form an ncRNA/RBP/histone repression complex with m^6^A-dependence, which can enhance the immune therapeutics effect of ICI, suggesting a promising therapeutic target.

**Figure:**
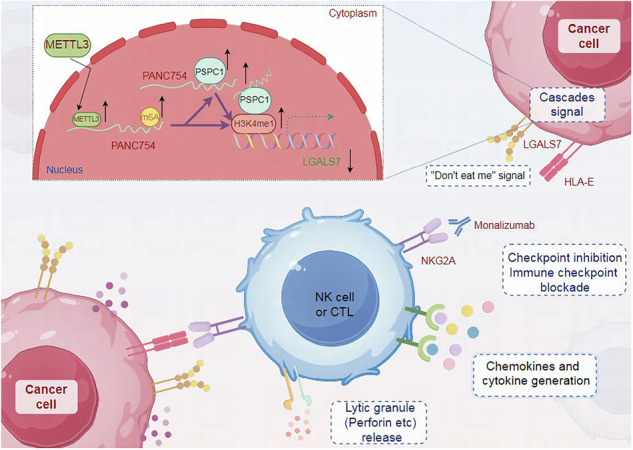
PANC754 is a novel pan-tumor suppressing non-coding RNA which is m6A-dependent and regulated by METTL3 modification enzyme. PANC754 is located at cellular nuclear and interacts with its RNA binding protein (RBP) PSPC1 and chromatin accessible histone H3K4me1, then can enhance immunotherapy capability of ICB anti-NKG2A against colorectal cancer through the immune evasive molecule LGALS7 signaling. Effect is that novel nuclear PANC754/PSPC1/H3K4me1 repression complex down-regulates the level “Don’t eat me” signal LGALS7 to improve the immune efficiency of ICB and induce NK or CTL cell to release perforin and cytokine to kill tumor cells. (Created by Figdraw).

PANC754 is a novel pan-tumor suppressing non-coding RNA which is m6A-dependent and regulated by METTL3 modification enzyme. PANC754 is located at cellular nuclear and interacts with its RNA binding protein (RBP) PSPC1 and chromatin accessible histone H3K4me1, then can enhance immunotherapy capability of ICB anti-NKG2A against colorectal cancer through the immune evasive molecule LGALS7 signaling. Effect is that novel nuclear PANC754/PSPC1/H3K4me1 repression complex down-regulates the level “Don’t eat me” signal LGALS7 to improve the immune efficiency of ICB and induce NK or CTL cell to release perforin and cytokine to kill tumor cells. (Created by Figdraw).

## Introduction

Non-coding RNAs (ncRNAs) are gene transcripts without classical protein-coding potential [1, 2]. Evidence extrapolated from whole genome sequencing (WGS) research suggests that no ≤2% of the human genome encodes proteins, while ~75% of the human genome is transcribed into ncRNAs [3]. There are many types of ncRNAs, including transfer RNAs (tRNAs) and ribosomal RNA (rRNA), small nucleolar RNAs (snoRNAs), microRNAs (miRNAs) and long non-coding RNAs (lncRNAs) [4, 5]. In our previous research, we found a novel pan-cancer down-expressed ncRNA, *PANC754* (https://pss-system.cponline.cnipa.gov.cn/documents/detail?prevPageTit=changgui). However, its function remains fairly obscure. The promoter regions of genes, particularly those involved in immune invasion, such as Galectin-7 (*LGALS7*) [6], harbor intricate and sophisticated regulatory networks [7], yet our understanding of their regulatory mechanisms is still constrained.

Since entering the 21st century, malignant tumors are still the second largest killer of human health [8], of which colorectal cancer (CRC) occupies the second place in gastrointestinal tumors [9]. There are two primary types of genes involved in tumorigenesis, oncogenes and tumor suppressor genes (TSGs) [8, 10, 11]. Classical TSGs are mainly focused on protein-coding genes, but with the development of technologies such as exon sequencing, it has become increasingly difficult to find novel TSGs in coding regions [12]. However, increasing evidence indicates that ncRNAs also have important regulatory functions similar to traditional oncogenes or tumor suppressor genes [13].

Therefore, in this study, we hypothesize that *PANC754* serves as a tumor suppressor ncRNA in CRC. We aim to establish its tumor-suppressive role through a series of in vitro and in vivo experiments and elucidate the underlying signaling pathways. Additionally, we will employ a co-culture system to assess its potential to enhance the efficacy of anti-NKG2A immunotherapy.

## Methods and materials

### Cell lines, plasmids, and patients

CRC SW480, Caco2, HCT116, and DLD1 cell lines were preserved by our laboratory and were cultured in Dulbecco’s Modified Eagle Medium or RPMI 1640 (KeyGEN Biotech, China) with 10% fetal bovine serum (FBS; ExCell, USA) and 1% antibiotic solution (penicillin-streptomycin, 10,000 U/mL) at 37°C in a humidified atmosphere containing 5% CO_2_. Overexpression of PANC754, METTL3, and PSPC1 was synthesized by GenScript Biotech Corp. (Nanjing, China) and cloned in pcDNA3.1 plasmid vector, then transfected with Lipofectamine 3000 transfection agent (Invitrogen, USA) into the above-mentioned cells.

26 CRC patients were cross-identified by two experienced pathologists and enrolled for sample collection from the Department of General Surgery in Affiliated Hospital of Nantong University, Jiangsu Province, China (The clinical information was shown in Table S1). The study was approved by the Institutional Review Board of Affiliated Hospital of Nantong University (No. 2018-K008). Written informed consent was obtained from each participant prior to sample collection.

### RT-PCR and real-time qPCR

Total RNAs were harvested using TRIzol (Invitrogen, USA) and converted to cDNA using the First-Strand cDNA Synthesis Kit (Vazyme Biotech, China) according to the manufacturer’s instructions. Real-time qPCR was performed by SYBR Green Master Mix (Vazyme Biotech) in an ABI 7500 PCR instrument (ABI, USA). As discussed previously, *GAPDH* gene was a better internal reference for ncRNA detection [14]. Therefore, *GAPDH* gene served as an internal reference in our study. Primer sequences were shown in Table S2.

### Determination of cell migration by wound healing repair assay

Cells were seeded into 6-well tissue culture plates at a density of ~70–80% monolayer confluence after 24 h of growth. The monolayer was gently and slowly scratched with a 200 µL pipette tip across the center of the well. Then another was scratched straight line perpendicular to the first line to create a cross in each well. After scratching, the well was gently washed twice with medium to remove the detached cells and the gap distance measured at 0 h. To determine the degree of wound healing, the gap distance remaining was measured at 24 h and 48 h post-scratch. Migration distance was calculated as follows: Migration distance = gap distance at 0 h−gap distance at t time (*t* = 24 h or 48 h) [15].

### MeRIP-PCR

The m^6^A methylation RNA immunoprecipitation (meRIP)-PCR kit was purchased from Millipore, USA. As the reagent supplier’s instruction, briefly, 3 µg total RNA was separated from CRC cells and purified with RNA purification kit (Invitrogen,USA). After fragmentation of RNA, anti-m^6^A antibody (Millipore, #17-10499) was used to enrich meRNA-protein complex, PCR was performed to amplify the RNA with m^6^A modification. meRIP-PCR primer sequences were shown in Table S3.

### shRNAs and siRNAs

Human shRNAs and siRNAs for METTL3, PANC754, and PSPC1 were synthesized by Gene PharmaInc. or Gene Adv Inc. (Shanghai, China), respectively. To avoid off-target effect, at least two pairs shRNAs and siRNAs of each target gene were utilized, and both of them knockdowned their targeted genes but did not impact them RNA levels of the other genes. siRNAs and shRNA core sequences of them were shown in Table S4.

### Cell line-derived xenograft (CDX) animal model

Fifteen 5-week-old female BALB/c nude mice were randomly divided into three groups. To establish tumor xenografts, HCT116 CRC cells (2 × 10^6^ cells/mouse/injection) mixed with Matrigel were subcutaneously injected into the armpit of each mouse for 2 consecutive days, totaling two injections. Subsequently, lentivirus expressing *PANC754* or negative control RNA was injected into the armpits of the mice to construct CDX models. Tumor growth and body weight were monitored weekly for at least five weeks. The animal experiments were ethically inspected and approved by the Laboratory Animal Ethical Committee of Nantong University (No. S20210301-020).

### RNA sequencing of PANC754 overexpression

RNA was extracted from CRC cells, and polyA selection was performed to enrich for mRNA, which was used to construct the cDNA library with a KAPA RNA Hyper Prep kit (Kapa, USA) according to the manufacturer’s instructions. The cDNA libraries were sequenced using Illumina HiSeq X 10 systems (Illumina, USA), achieving an average sequencing depth of 140 X per fragment. The transcriptome data were analyzed with the Tophat2 and Cufflinks pipeline, adhering to the provided instructions.

### Immunohistochemical staining (IHC) and immunocytochemical staining (ICC)

CRC mouse tissues were fixed in formalin and embedded in paraffin for sectioning. Sections of 4–5 μm thickness are cut and mounted on slides, or the CRC cultured cells were mounted on a slide, then deparaffinized by immersing in xylene or graded alcohols and rehydrated in a descending series of alcohols. After antigen retrieval, sections were blocked with serum or bovine serum albumin (BSA) to reduce non-specific binding of antibodies. The primary Ki67 (Abmart, #TW0001), MMP9 (Cellsignal, #14472), and E-cadherin (Cellsignal, #13667) antibodies were added to the sections and incubated for 1–2 h at room temperature, then secondary antibody incubation for 30 min at room temperature. The antibody-antigen complex is visualized by adding substrate solution diaminobenzidine (DAB) for peroxidase conjugate to the sections and incubating for a few minutes. The substrate solution develops a brown color in the presence of peroxidase, indicating positive staining.

### Western blotting

Nuclear and total proteins were extracted by nucleated and total protein extraction kit (Beyotime, China), including PMSF proteinase inhibitor (Beyotime) and quantitated by BCA protein concentration assay kit (Beyotime). 1 µg of nuclear protein or total proteins from each group were separated by 10% sodium dodecyl sulfate/polyacrylamide gel electrophoresis (SDS-PAGE) and transferred to 0.45 μm polyvinylidene fluoride (PVDF) membrane (Millipore, USA). Following blocking with 5% non-fat milk (Beyotime), membranes were incubated with anti-PSPC1 (Proteintech,China; #16714-1-AP), anti-LGALS7 (Abcam,UK; #ab206435), anti-METTL3 (ABclonal,USA; #A8370), anti-H3K4me1 (Abcam, #ab176877), anti-H3K27ac (Abcam, #ab177178), anti-PCNA (Cellsignal, #13110), anti-Histone H3 (Cellsignal, #4499), anti-N-cadherin (MCE, #HY-P80761), anti-Vimentin (MCE, #HY-P80371), anti-SNAIL (MCE, #HY-P81135), and anti-GAPDH (ABclonal, #AC002) overnight at 4°C and then incubated with HRP-conjugated secondary antibodies (Abcam, USA) for 1 h at room temperature. Finally, protein bands were detected using enhanced chemiluminescence (ECL) detection kit (Beyotime, China) and developed on x-ray film (Carestream, USA) in the darkroom. The band gray-intensities were analyzed via Gel-Pro Anazyler 3.0 software with the default settings. Histone H3 and GAPDH were used as the loading control for nuclear protein and cytoplasma protein normalization, respectively.

### Fluorescence in situ hybridization

For the fluorescence in situ hybridization (FISH) assay, we utilized a set of pre-labeled short DNA oligonucleotides, each about 20 nucleotides long, to create a probe panel consisting of up to 48 independent probes. These probes collectively bind to the target RNA *PANC754*, producing a strong fluorescent signal that illuminates RNA or individual RNA molecule clusters as distinct punctate spots without requiring enzymatic signal amplification. Adhering to the protocol provided by Genecreate (China), HCT116 cells were fixed with 4% paraformaldehyde at room temperature for 10 min. Both the target RNA probe for PANC754 and the reference probes (U6 for nuclear and 18S for cytoplasmic localization) were prepared in hybridization solution and hybridized with the cells overnight at 37°C in the dark. Subsequent to a 10-minute DAPI staining at room temperature, the cells were examined using laser confocal microscopy to visualize the FISH signals.

### RNA pulldown

To identify proteins interacting with *PANC754*, we conducted an RNA pulldown assay using a kit from Genecreate lnc. (Wuhan, China). The procedure was as follows: the *PANC754*-specific probe was labeled with a biotin tag. This labeled RNA was then incubated with streptavidin magnetic beads to allow for the binding of proteins interacting with the RNA. After incubation, the beads were washed to remove non-specific interactions, and the RNA-protein complexes were eluted. The eluate was analyzed to identify candidate RNA-binding proteins (RBPs) that interact with PANC754, following the manufacturer’s instructions. The sense and antisense chain of PCR primers for the PANC754 DNA template were shown in Table S5.

### LC-MS/MS

Following the purification of proteins from the RNA pulldown, we conducted liquid chromatography (LC) - tandem mass spectrometry (MS/MS) analysis. The procedure was as follows: target protein bands were first excised from two-dimensional electrophoresis gels. These bands were then treated with a final concentration of 10 mM dithiothreitol (DTT) to reduce disulfide bonds, followed by alkylation with 5 mM iodoacetamide (IAM) to prevent reformation. Enzymatic digestion was performed using 1 μg of trypsin. The resulting peptide fragments were analyzed using LC-MS/MS (ekspert^TM^ nanoLC; AB Sciex TripleTOF 5600-plus) to determine charge states and generate mass spectra. Protein identification was carried out using ProteinPilot software with a confidence threshold set at Conf ≥95%. Common contaminant proteins, including keratins, antibodies, and serum albumin, were excluded from the analysis (Proteins exclusively binding to the sense strand of PANC754 are presented in Table S6). This approach facilitated the definitive identification of the target protein from the mass spectrometry data.

### Co-culture of CRC cells and PBMCs

Carboxyfluorescein diacetate succinimidyl ester (CFDA-SE) was utilized to label CRC HCT116 cells and peripheral blood mononuclear cells (PBMCs) derived from healthy donors. These cells were then co-cultured at a ratio of 5:1 in the presence of the cytokine interleukin-2 (IL-2; R&D Systems, USA) to stimulate the cells. Monalizumab, a monoclonal antibody that inhibits NKG2A (MCE, China), and overexpression plasmids of PANC754 (OE-PANC754) or empty plasmids (Control) were introduced into the co-culture system. After a 48-hour incubation period, both the cells and the culture supernatant were harvested separately for subsequent analysis.

### Statistical analysis

High-throughput differential gene expression and meta-analysis were conducted under R (v3.5.1). All data from functional experiments were shown as the mean ± standard deviation. Statistical significance was defined as *P* < 0.05, with analyses performed using SPSS 20.0 software. For comparisons between two groups, a two-tailed t-test was applied, while for comparisons involving three or more groups, One-Way ANOVA (analysis of variance) was utilized.

## Results

### PANC754 inhibits cell growth and metastasis of CRC cell lines

**Fig. 1:**
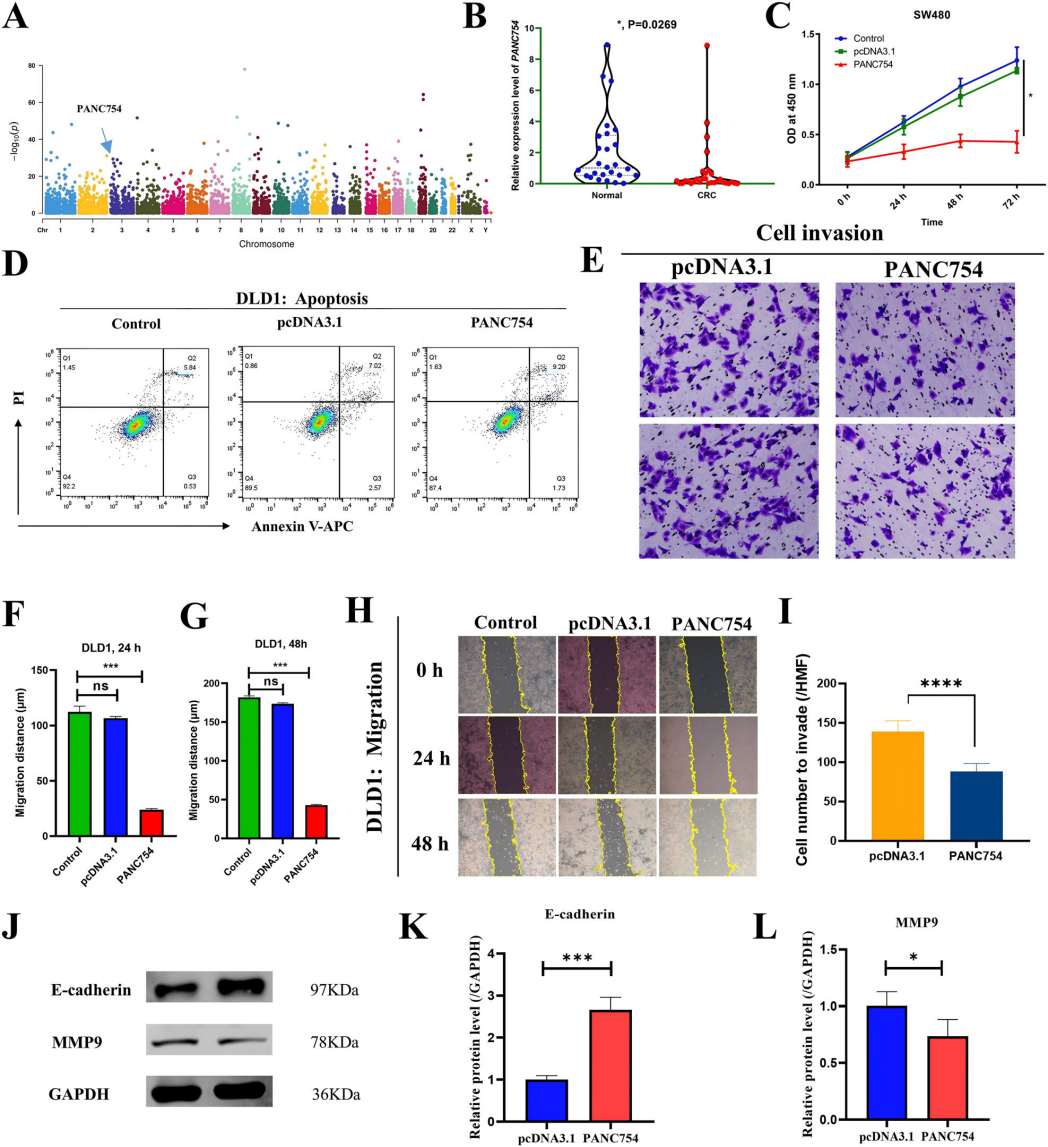
*PANC754* markedly inhibited the cell growth and metastasis of CRC cell lines. **A** Manhattan map of PANC754 crosses 23 types of human cancers. Cancer samples were collected from the TCGA project (*N* = 10,490). Gene expression level was log2 transformed before the meta-analysis. Random effect models were applied for the aggregation. **B** The expression levels of PANC754 were validated in the tumor tissues and paratumorous tissues of 26 pairs of colorectal cancer patients by quantitative PCR. **C** The proliferative curve of SW480 cell line with overexpression of PANC754 by CCK-8 assay. Control, untransfected SW480; pcDNA3.1, empty pcDNA3.1 plasmid transfected; PANC754, overexpression plasmid of PANC754 transfected; *P < 0.05. **D** Cell apoptosis detection by flow cytometry. Each experiment was repeated at least three times. **E**–**G** Determination of cell migration in DLD cell line by wound healing repair assay and its statistical histograms in 24 h F or 48 h. **G** ns, no significance; ***P < 0.001. **H**, **I** Cell invasion detection by transwell chamber assay and their statistical histograms in SW480 cell line. ****P < 0.0001. **J**–**L** MMP9 and EMT marker E-cadherin were detected by WB and their statistical histograms in the SW480 cell line. Each experiment was repeated at least three times.

Using a random effects model, we identified a novel ubiquitously downregulated ncRNA named *PANC754* from 1615 ncRNAs that were significantly upregulated or downregulated (Fig. 1A, Bonferroni-corrected *P* < 0.05 with a Manhattan map). Our CRC cohort also validated that *PANC754* was significantly low expression state in CRC than that in paracancerous normal tissues (Fig. 1B).

To investigate the functional role of *PANC754* in cancer cell phenotypes, tumor cell lines were transfected with a plasmid containing *PANC754* and analyzed by CCK-8 assay and so on. The overexpression efficacy of *PANC754* was validated (Fig. S1–1A). Compared to the control (untransfected) or pcDNA3.1 (transfected with empty vector/pcDNA3.1 plasmid) groups, the growth ability of SW480 cell line and DLD1 cells transfected with pcDNA3.1-*PANC754* were gradually inhibited, especially after 48 h and 72 h in culture (*P* = 0.0004 and *P* = 0.0016, respectively; Fig. 1C, Supplementary Fig. S1–D).

In order to determine whether overexpression of *PANC754* can induce tumor cell apoptosis, the rates of apoptosis were measured by flow cytometry. As shown in Fig. 1D, the apoptosis rate of DLD1 cell line, was significantly increased following *PANC754* transfection compared to the rates of the control and pcDNA3.1 groups (*P* < 1.0 × 10^−4^). The same effect was observed in SW480 cells (Fig. S1–1C). Through a scratch assay experiment, as shown in Fig. 1E–G, the gap distances in the transfected pcDNA3.1-*PANC754* group (abbreviated as *PANC754*) of CRC cells after culturing for 24 h and 48 h were significantly wider than those in the control and pcDNA3.1 groups with *P* < 1 × 10^−4^ and *P* < 1 × 10^−4^, respectively (Fig. S1–2A–C). A transwell chamber experiment was also performed to determine the effect of *PANC754* overexpression on invasion of CRC. After 48 h culture of SW480 cells, the *PANC754* group was observed to have fewer cells invading the membrane of the transwell chamber compared to the pcDNA3.1 groups (*P* < 0.0001; Fig. 1H, I). Furthermore, in SW480 cells of the *PANC754* group, endothelial-mesenchymal transition (EMT) biomarkers exhibited significant upregulation of E-cadherin and downregulation of N-cadherin, and SNAIL compared to the control group (Fig. 1J–L; Fig. S1–2E). Together, PANC754 was a suppressor ncRNA to CRC.

### PANC754 inhibits CRC cell proliferation and metastasis in vivo

**Fig. 2:**
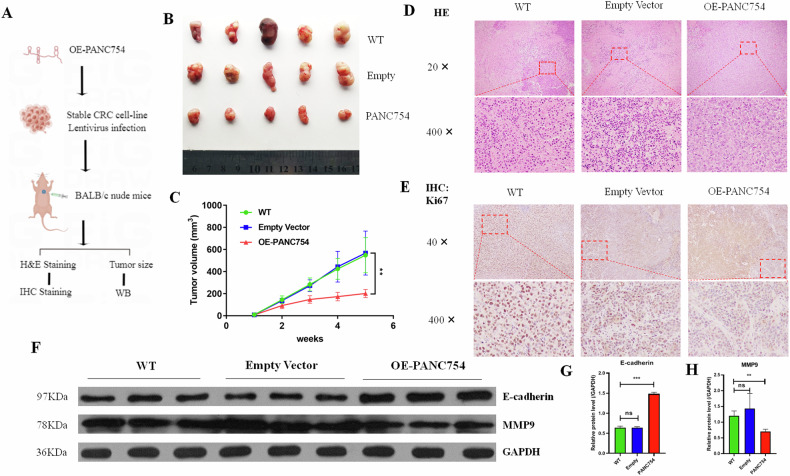
*PANC754* significantly suppressed cell growth and metastasis in CDX model. **A** The pipeline of CRC HCT116 cell line-derived xenograft (CDX; Created by Figdraw). **B** The presentation of the CDX tumor sizes in three different treatment groups (*n* = 5, respectively). WT, lentivirus uninfected SW480; Empty, empty lentivirus-infected; PANC754, overexpression PANC754 lentivirus-infected. **C** The CDX tumor volumes in three treated CDX groups. Empty vector, empty lentivirus vector infected; OE-PANC754, overexpression PANC754 lentivirus vector infected. **P < 0.01. **D** The representative image of H&E staining in three treatment CDX groups. **E** The representative image of IHC staining of the proliferation marker Ki67 in three different treatment groups. **F**–**H** The protein levels of the metastasis biomarkers MMP9 and E-cadherin in CDX were detected by WB and their statistical histograms. ***P < 0.001.

To further explore the function of PANC754 in CRC, we established nude mouse xenograft models (CDX [15]; Fig. S2A, B) by subcutaneously injecting CRC cells transfected with lentivirus expressing PANC754 or negative control RNA into the flanks of nude mice (Fig. 2A). Results showed that xenograft tumors formed by CRC cells transfected with PANC754 grew significantly slower than those transfected with negative control RNA (Fig. 2B, C). HE staining further confirmed that PANC754 suppressed CRC tumorgenesis (Fig. 2D).

IHC staining with Ki67 antibody revealed that the number of Ki67-positive cells was significantly lower in the PANC754 overexpression group than in the control group (Fig. 2E). In addition, WB analysis demonstrated that overexpression of PANC754 suppressed MMP9 expression and enhanced E-cadherin expression in xenograft tumors (Fig. 2F–H). These results suggest that PANC754 inhibits CRC cell proliferation and metastasis in vivo by regulating Ki67, MMP9, and E-cadherin.

### PANC754 expression is regulated by m^6^A modification

**Fig. 3:**
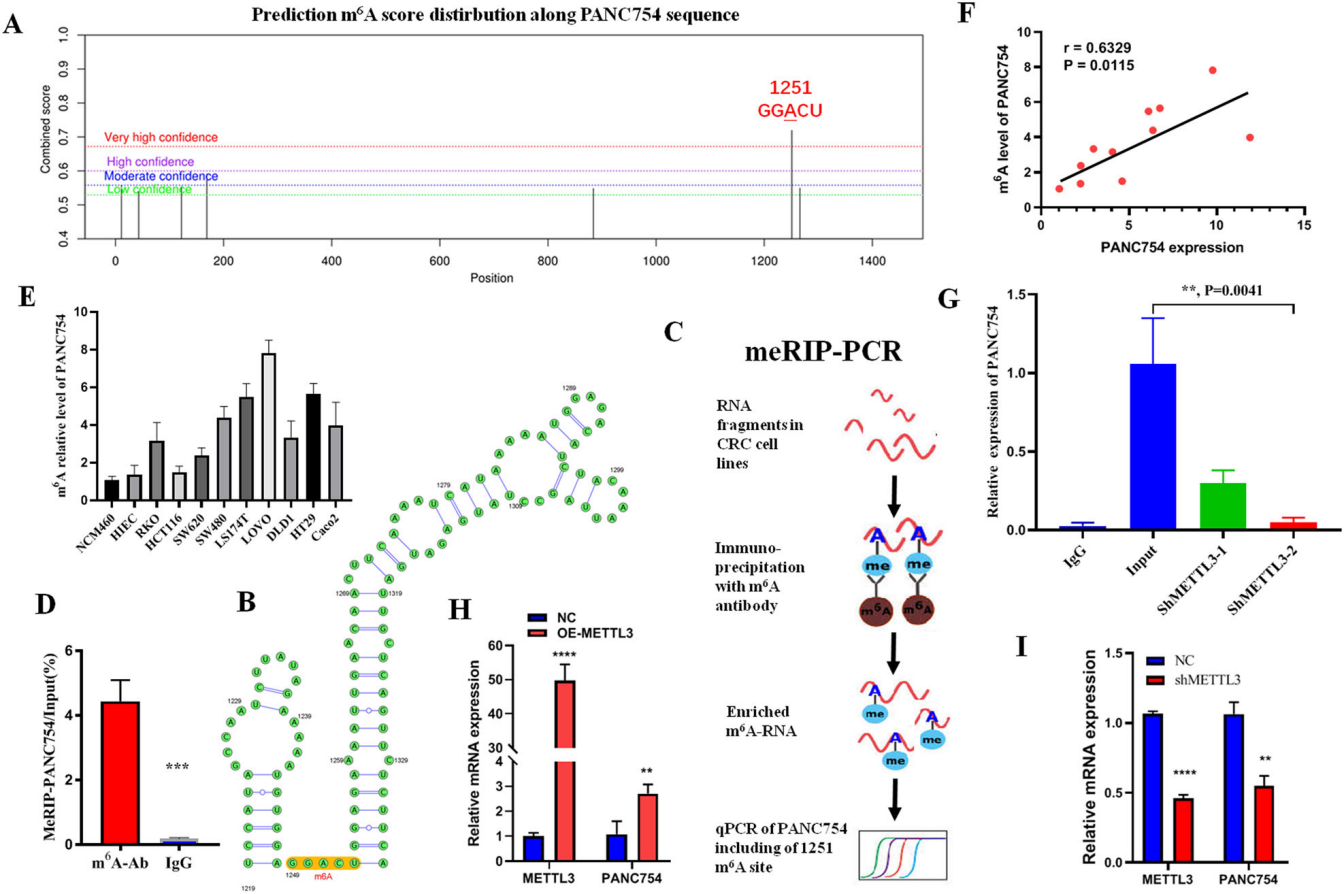
*PANC754* was regulated by m^6^A modification with methyltransferase METTL3. **A** Prediction of m^6^A site of *PANC754* in the SRAMP online website. High combined score implies high confidence. **B** Prediction of *PANC754* secondary structure including m^6^A site in the SRAMP online website. **C** The schematic diagram of meRIP-PCR assay (Created with Microsoft PPT, and some icons in the schematic are sourced from bersinbio.com). **D** The m^6^A level of *PANC754* was detected by meRIP-PCR. IgG served as a negative control. ***P < 0.001. **E** The m^6^A level of *PANC754* in various CRC cell lines by meRIP-PCR. **F** The positive correlation between the m^6^A level and the mRNA level of *PANC754*. **G** With the knockdown of methyltransferase METTL3, the m^6^A level of *PANC754* was markedly decline. **H**, **I** The mRNA level of PANC754 was significantly increase (**H**) or significantly decrease (**I**) after the overexpression or down-regulation of METTL3. NC, empty vector or scramble shRNA vector control; OE-METTL3, overexpression of METTL3; shMETTL3, down-regulation of METTL3; **P < 0.01; ****P < 0.0001; ns no significance; Each experiment was repeated at least three times.

Mounting studies have indicated that m^6^A modification can regulate the expression of ncRNA, thereby influencing its function [16, 17]. Utilizing the SRAMP online portal, we identified a potential m^6^A modification site, GGACU, within the PANC754 sequence at positions 1249–1253 (Figs. 3A, B and S3A). To confirm m^6^A modification at the 1251 A site of PANC754, we conducted meRIP-PCR analysis (Fig. 3C). Our results showed a higher peak for PANC754 m^6^A compared to the IgG control in CRC cell line (Figs. 3D and S3B). Additionally, we observed that CRC cell lines exhibited a higher level of m^6^A modification than the normal intestinal epithelial cell line NCM460 (Fig. 3E). Moreover, the m^6^A level was positively correlated with the mRNA expression of PANC754 (Fig. 3F).

To further investigate the role of m^6^A in regulating PANC754, we manipulated the expression of METTL3, a key methyltransferase enzyme of m^6^A modification [18, 19]. We discovered that knocking down METTL3 led to a significant decrease in PANC754’s m^6^A modification compared to that in the input control (Fig. 3G). Concurrently, METTL3 knockdown markedly reduced *PANC754* mRNA levels (Fig. 3I), while METTL3 overexpression significantly increased its expression (Fig. 3H). Collectively, these findings indicate that *PANC754* expression is regulated by METTL3 in a m^6^A-dependent manner.

### The nuclear-located PANC754 binds with its RBP PSPC1

**Fig. 4:**
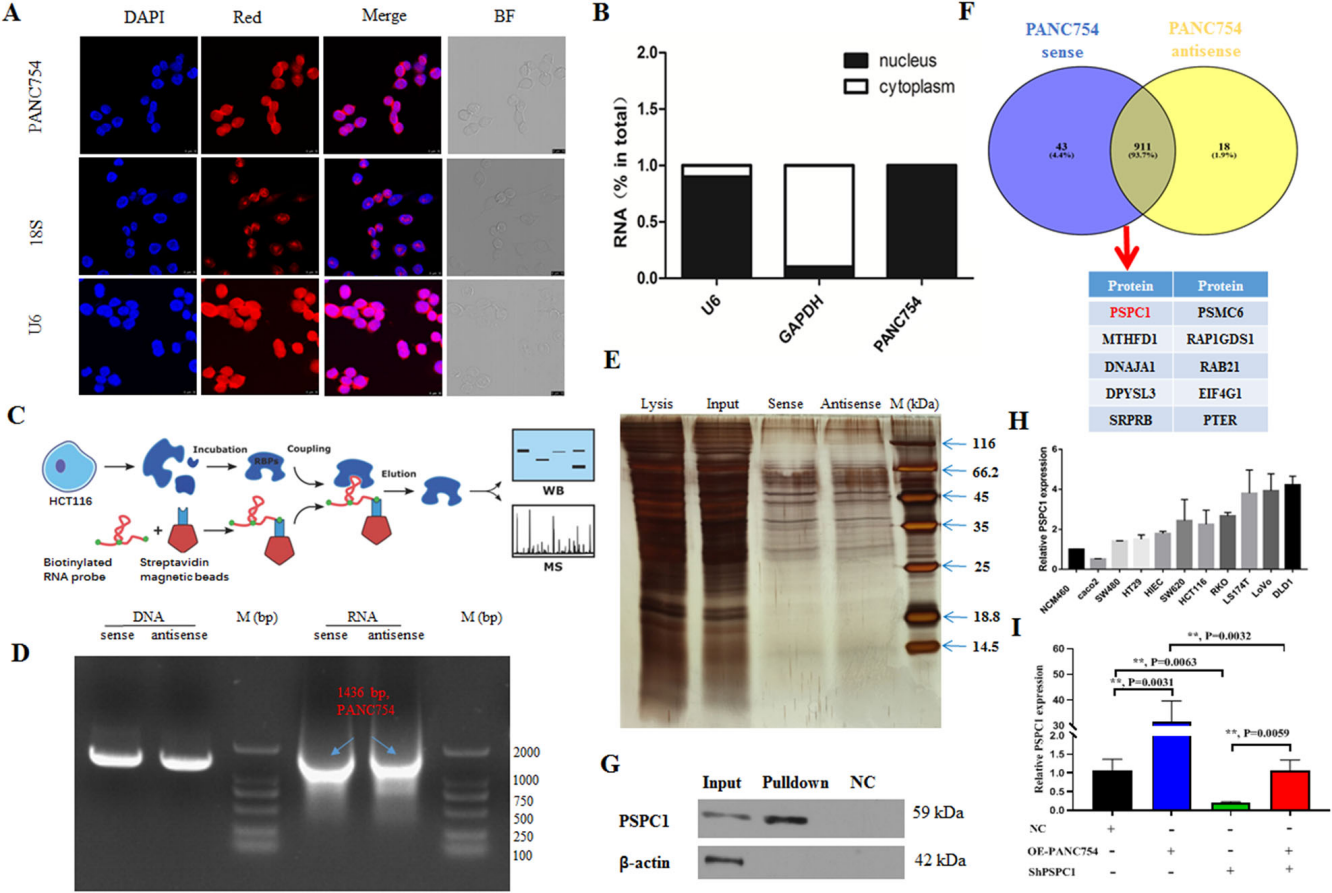
*PANC754* was the nuclear-located and bound with its RBP PSPC1. **A** The subcellular location of *PANC754* was detected by RNA FISH. U6 served as a nucleus location control; 18S served as a cytoplasm location biomarker. **B** The subcellular location of *PANC754* by the nuclear-cytoplasmic fractionation experiment. GAPDH served as a cytoplasmic location biomarker. **C** The flowchart of RNA pulldown of PANC754 and subsequent analysis (Created with Microsoft PPT, and some icons in the schematic are sourced from bersinbio.com). **D** The agarose gel electrophoresis map verified that in vitro transcription approach to produce both sense and antisense RNA transcripts of *PANC754*. **E** The silver staining pattern of RNA pulldown between sense and antisense RNA transcripts of *PANC754*. **F** The Venn diagram of LS-MS/MS analysis between the sense and antisense RNA transcripts of *PANC754*. **G** Immunoblotting of the RNA pulldown samples further confirmed PSPC1 as the RBP for *PANC754*. **H** The mRNA level of PSPC1 in various CRC cell lines. **I** The mRNA level of PSPC1 when overexpression of *PANC754* and/or knockdown of PSPC1. NC, HCT116 cells with untransfected plasmid; OE-PANC754, overexpression PANC754 plasmid transfected; shPSPC1, shPSPC1 plasmid transfected.

As an ncRNA’s functional role is contingent upon its cellular localization [20]. Utilizing FISH and a nuclear-cytoplasmic separation assay, we established that PANC754 resides in the nucleus of CRC cells (Fig. 4A, B). Given its nuclear presence, the function of PANC754 is likely mediated by its associated RBPs [20]. We systemic screening its RBP by employing an in vitro transcription approach to produce both sense and antisense RNA transcripts of *PANC754* (Fig. 4D). RNA pulldown assays, followed by silver staining, revealed proteins that interact with the sense and antisense RNA of *PANC754* (Fig. 4C), with the results depicted in Fig. 4E. LC-MS/MS analysis identified 43 significantly different proteins binding to the sense RNA of PANC754, among which paraspeckle component 1 (PSPC1) stood out for its high abundance and unique peptide coverage (Figs. 4F and S4A, B; Table S6). Immunoblotting of the RNA pulldown samples further confirmed PSPC1 as an RBP [21] for *PANC754* (Fig. 4G), may bind to *PANC754* by its RNA recognition motif (Fig. S4C, D).

Consequently, we assessed the expression levels of PSPC1 (Fig. 4H) and PANC754 (Fig. S1–1B) across CRC cell lines and selected HCT116 cells to investigate the interaction between *PANC754* and PSPC1. As illustrated in Fig. 4I, overexpression of PANC754 significantly increased PSPC1 expression levels compared to those in the control group. However, when PANC754 was overexpressed alongside PSPC1 knockdown, the expression of PSPC1 was notably rescued. Together, these findings indicate that *PANC754* exerts its roles depending on its RBP PSPC1.

### PANC754 suppresses CRC progression via inhibiting the immune evasive molecule LGALS7

**Fig. 5:**
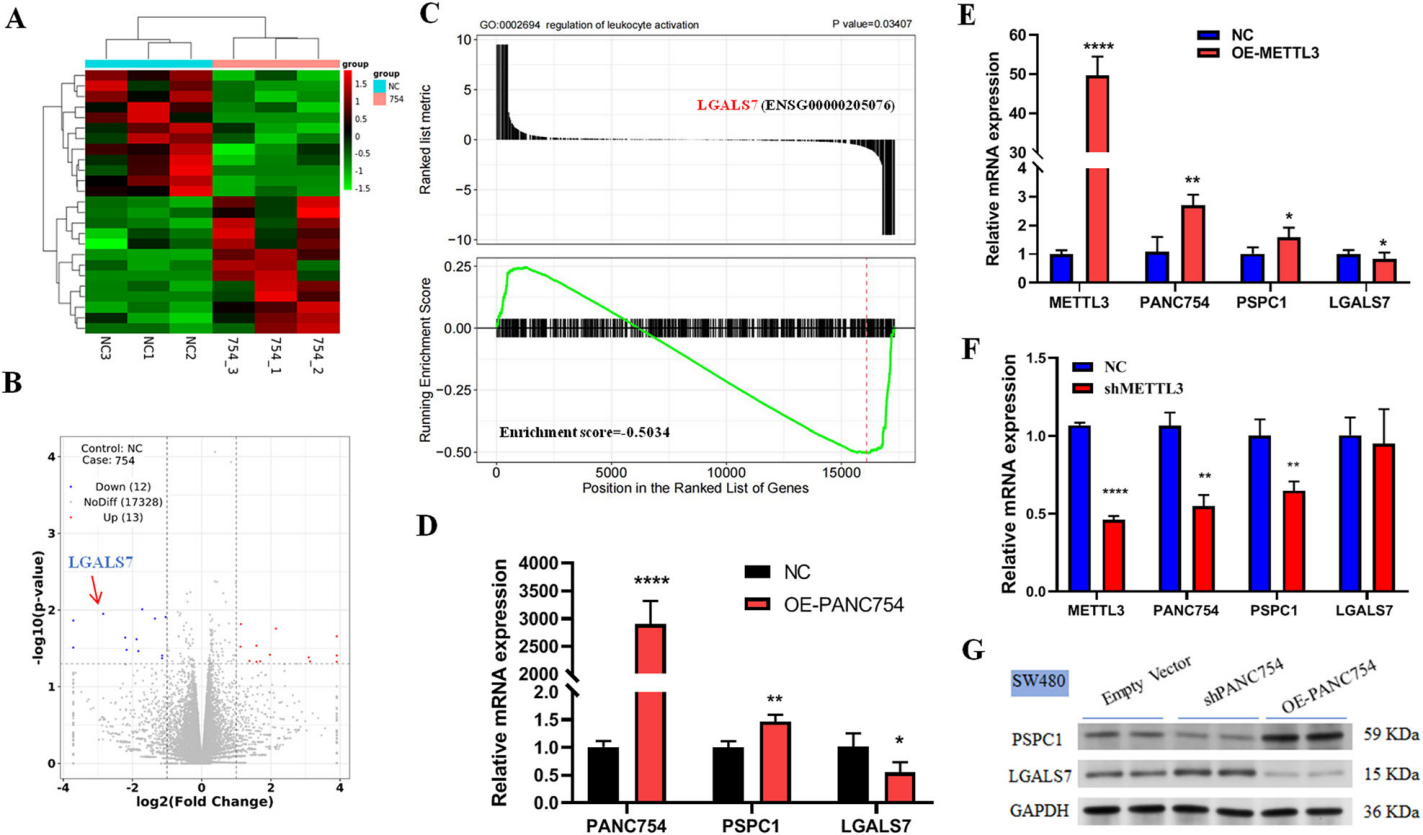
PANC754 binding with its RBP PSPC1 suppresses CRC progress via inhibiting immune evasive molecule LGALS7. **A** The heat map of *PANC754* overexpression in DLD1 cell line. NC, HCT116 cells with untransfected plasmid; 754, overexpression PANC754 plasmid transfected. **B** The volcano plot of *PANC754* overexpression, where the arrow pointed LGALS7 significantly downregulated. **C** GSEA (gene set enrichment analysis) indicated that PANC754 overexpression led to the enrichment of the immune signals of leukocyte acitvation. **D** The mRNA levels of *PANC754*, *PSPC1*, and *LGALS7* gene after PANC754 overexpression in DLD1 cells were detected by qRT-PCR. *P < 0.05; **P < 0.01; ***P < 0.001. Each experiment was repeated at least three times. **E** The mRNA levels of *METTL3*, *PANC754*, *PSPC1*, and *LGALS7* gene after METTL3 overexpression in HCT16 cells were detected by qRT-PCR. **F** The mRNA levels of *METTL3*, *PANC754*, *PSPC1*, and *LGALS7* gene after METTL3 knockdown in SW480 cells were detected by qRT-PCR. **G** The protein expression of PSPC1 and LGALS7 was determinedin SW480 cells by Western blotting. GAPDH served as the loading control; Empty vector, transfected with empty pcDNA3.1 plasmid group; OE-PANC754, transfected with pcDNA3.1-PANC754 overexpression plasmid group; shPANC754, transfected with shPANC754 kncokdown plasmid group.

We then performed RNA sequencing of *PANC754* overexpression to explore the signaling pathway of PANC754 against CRC. Galectin-7 (*LGALS7*) gene was found to significantly downregulated in *PANC754* overexpression CRC cells than that in the control cells (Figs. 5A, B and S6–1, 2). *LGALS7* gene is commonly served as a tag of “Don’t eat me” in fostering innate immune evasive programs [6, 22] and our data also confirmed that it significantly enriched in immune regulation process such as leukocyte activation (Fig. 5C). So, we detected the mRNA level and the protein level of *PSPC1*, *LGALS7*, *PANC754* genes after overexpression of PANC754 and METTL3 in turn. PANC754 significantly downregulated the expression of LGALS7 and prominently increased the expression of PSPC1 (Fig. 5D). In contrary, knockdown of PANC754 significantly increased the level of LGALS7 protein, while it markedly decreased the level of PSPC1 protein (Figs. 5G and S5). Furthermore, METTL3 overexpression had the similar tendency with PANC754 overexpression (Fig. 5E), indicated METTL3/PANC754 axis can suppress LGALS7 level. Loss of function with *METTL3* gene simultaneously markedly decreased the expression of PANC754 and PSPC1 genes (Fig. 5F).

Together, these suggest PANC754 may suppress CRC progression via inhibiting the expression of the immune evasive molecule LGALS7.

### Nuclear PANC754/PSPC1/H3K4me1 repression complex regulates LGALS7 expression

**Fig. 6:**
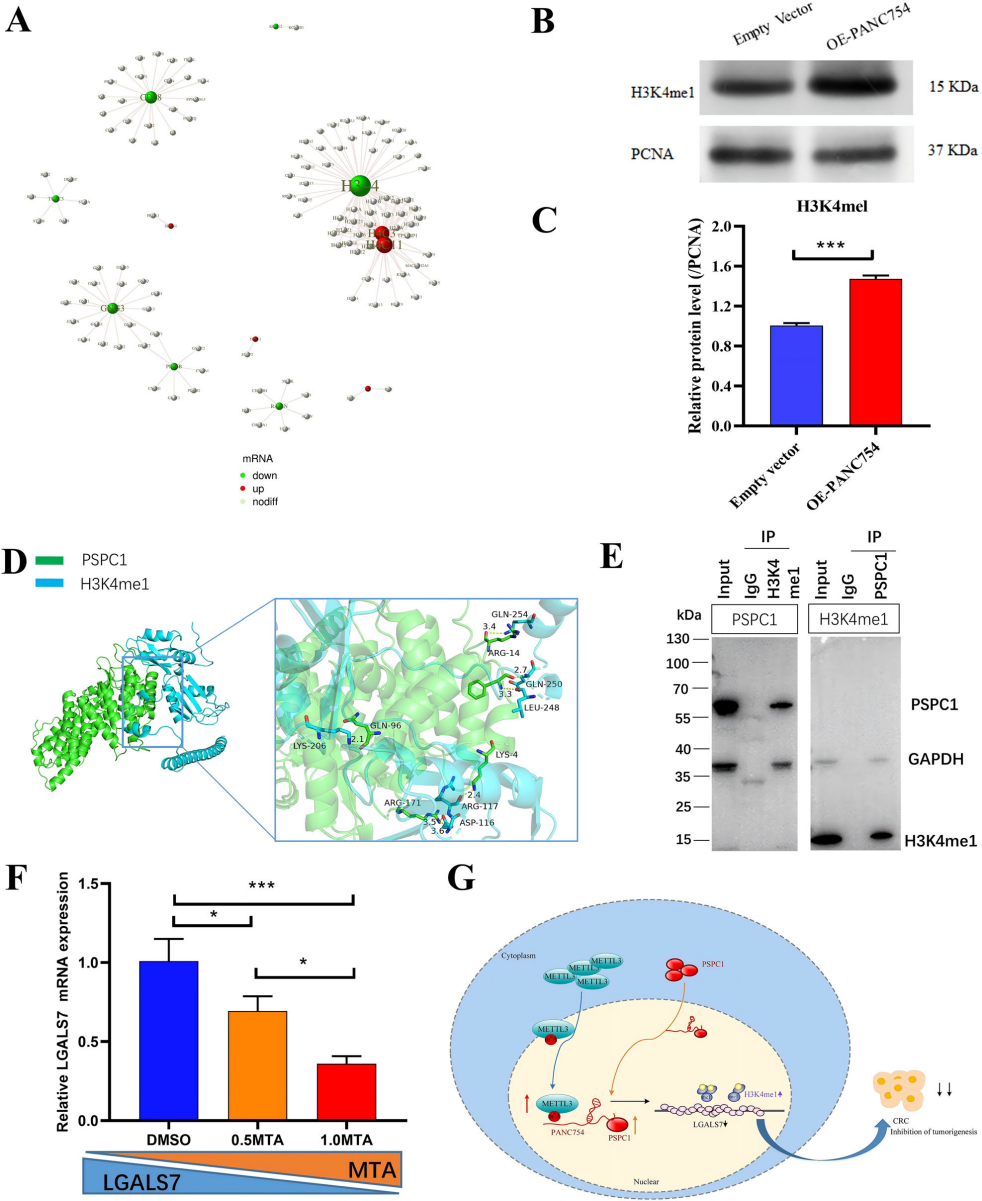
PANC754/PSPC1/H3K4me1 repression complex regulates LGALS7 expression. **A** The protein-protein interaction (PPI) diagram from RNA-Seq data suggests a strong correlation between PANC754’s downstream effects and the histone-encoding gene *H3-4*. **B, C** The histone H3K4me1 protein level after PANC754 overexpression in SW480 cells was detected WB and their statistic histograms. **C** PCNA was served a internal control. ***P < 0.001. Each experiment was repeated at least three times. **D** The interplay between PSPC1 and H3K4me1 by molecular docking experiment. **E** The direct interaction between PSPC1 and H3K4me1 was detected in Caco2 cells by Co-IP. **F** By using H3K4 inhibitor MTA, the mRNA level of *LGALS7* gene decreasing slowly company with the increasing concentration of MTA in DLD1 cells. **G** A schematic diagram showed the model of m^6^A-dependent nuclear ncRNA PANC754 coupled with its binding protein PSPC1 and chromatin-accessible H3K4me1 protein to form ncRNA/RBP/histone repression complex to downregulate the immune invasive LGALS7 signaling, inhibiting colorectal cancer progress (Created with Microsoft Visio).

PANC754 overexpression leads to LGALS7 downregulation; this regulation process may be influenced by other factors. To the end, we further mined our RNA-Seq data and protein-protein interaction (PPI) diagram suggests a strong correlation between PANC754’s downstream effects and the histone-encoding gene *H3-4* (Fig. 6A). Therefore, we searched the upstream regulation region of *LGALS7* gene, and found that H3K4me1 or H3K27ac can bind to its promoter region (Fig. S6–3A). Upregulated PANC754 significantly increased the level of H3K4me1 and H3K27ac (Fig. 6B, C), while the degree of increasing H3K4me1 was more than that of H3K27ac (Fig. S6–3D).

Due to PSPC1 is a RBP with transcription factor (TF) feature [23, 24], we speculated that it may interplay to H3K4me1 to negatively regulate LGALS7 level. To demonstrate this, we firstly predicted the interaction between them in GeneMENIA portal (Fig. S6–3B). Then we performed the molecular docking experiment to verify that PSPC1 can bind to H3K4me1 (Fig. 6D). Lastly, we utilized co-IP to further confirm that the direct interaction between them in HCT116 cells (Fig. 6E). Supplementary, by using H3K4 inhibitor MTA, we found that the mRNA level of *LGALS7* gene decreasing slowly company with the increasing concentration of MTA (Fig. 6F).

In brief, METTL3-modificatory m^6^A regulates the interaction of PANC754 with PSPC1 and H3K4me1 to form ncRNA/RBP/histone repression complex at gene promoter region to mediate the “Don’t eat me” LGALS7 signaling pathway, thereby exerting tumor-suppressing effects against CRC (Fig. 6G).

### Upregulation of the immunotherapeutic capability of Monalizumab by the combination of PANC754 full-length RNA delivery

As mentioned above, PANC754 upregulation can break down the immune evasion effect of LGALS7. “Don’t eat me” signal of LGALS7 main targets to natural killer cells (NK) or cytotoxic T lymphocytes (CTLs) [25]. And NKs or CTLs are tightly related to *NKG2A* gene [26, 27]. Consequently, the immune checkpoint blockage (ICB) of NKG2A gene, Monalizumab [28] combined with PANC754 full-length overexpression was applied to the immune therapy of CRC in our translational medical research.

**Fig. 7:**
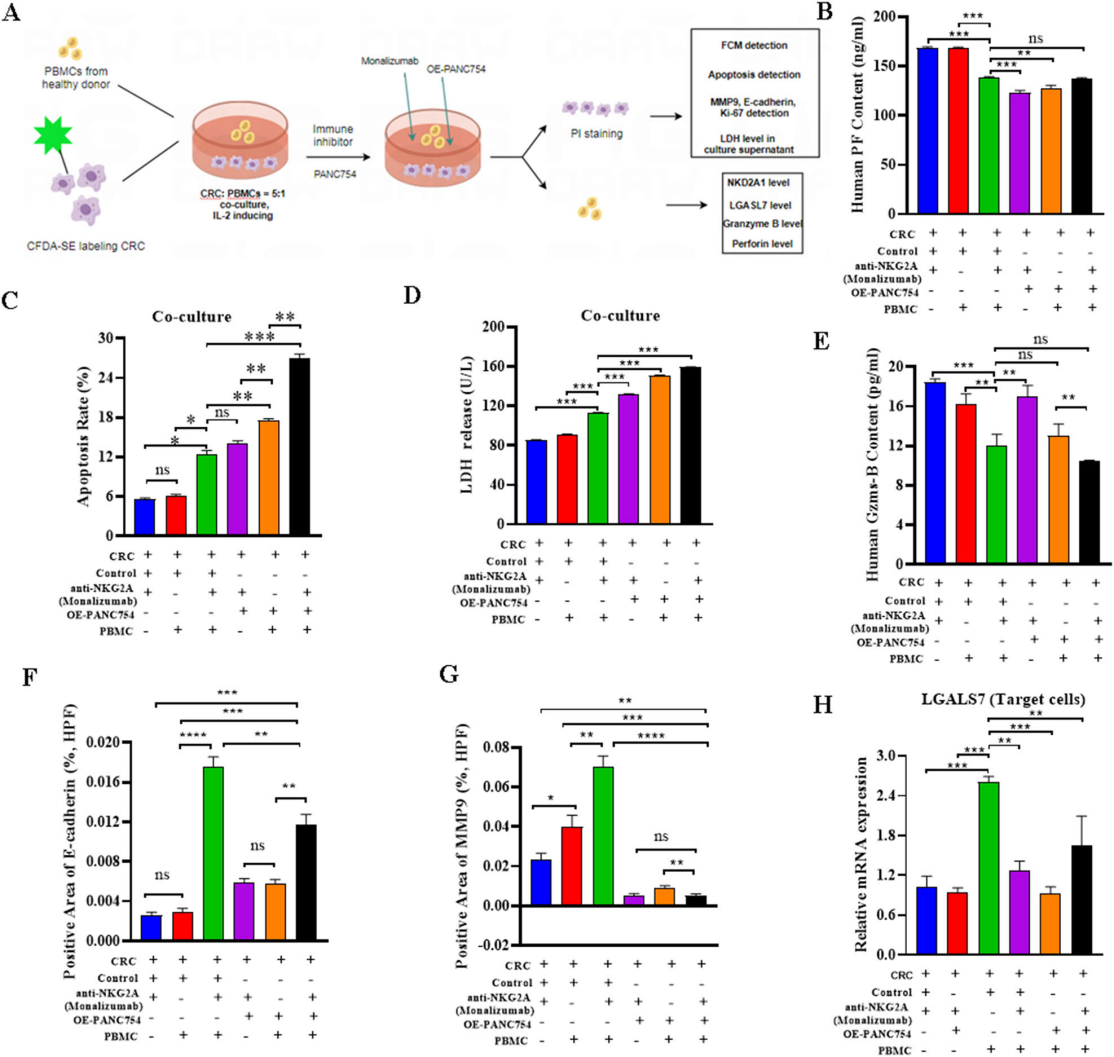
Upregulation of the immunotherapeutic ability of Monalizumab by PANC754 combination. **A** The flowchart of co-culture system of PANC754 and the immune checkpoint blockage (ICB) of *NKG2A* gene, Monalizumab (Created by Figdraw). **B** The Perforin content in the culture supernatant of co-culture system was detected by ELISA. PF, Perforin; CRC, HCT116 cells; Control, transfected with empty plasmids; PBMC, peripheral blood mononuclear cells; OE-PANC754, transfected with *PANC754* overexpression plasmid. **C** The apoptosis rate of HCT116 cells was detected by FCM. *P < 0.05; **P < 0.01; ***P < 0.001; ns, no significance. **D** The LDH concentration in the culture supernatant detected by velocity method in the biochemical analyzer. **E** The LDH concentration in the culture supernatant detected by ELISA. Gzms-B, granzyme B. **F** E-cadherin protein level in HCT116 cells was determined by immunocytochemical (ICC) staining. **G** The positive rate of MMP9 protein in HCT116 cells was determined by ICC staining. **H** The mRNA level of *LGALS7* gene in HCT116 cells was determined by qRT-PCR. ****P < 0.0001. Each experiment was repeated at least three times.

Leveraging co-culture cell system, we determined the generation of inflammatory cytokine, chemokine and the release of lytic granule from peripheral blood mononuclear cells (PBMCs) and HCT116 cells (Fig. 7A). Compared to the sole use of Monalizumab or PANC754, the combined treatments of Monalizumab and PANC754 significantly increased the apoptosis rate (Figs. 7C and S7–1A) and the lactate dehydrogenase (LDH) release in CRC cells (Fig. 7D). Conversely, the release of Perforin [29] by the immune cells was significantly accumulated (Fig. 7B), while Granzyme B (GZMB) [30] showed no significance (Fig. 7E). The expression of *NKG2A* on CRC cells significantly increased compared to the control group, while the difference in NKG2D [31] was not significant, and LGALS7 expression was significantly suppressed (Fig. S7–1).

Using immunocytochemical staining, we found that compared to the individual use of Monalizumab or PANC754, the combination therapy of Monalizumab and PANC754 significantly decreased the level of MMP9 [15], a marker of cellular migration (Figs. 7G and S7–2B), and significantly increased the level of *E-cadherin* gene [15], a biomarker of epithelial-mesenchymal transition (Figs. 7F and S7–2A).

Together, these results validated that the immunotherapeutic ability is significantly upregulated through the use of Monalizumab and PANC754 combination.

### cfPANC754 may be a promising biomarker for the diagnosis of CRC

**Fig. 8:**
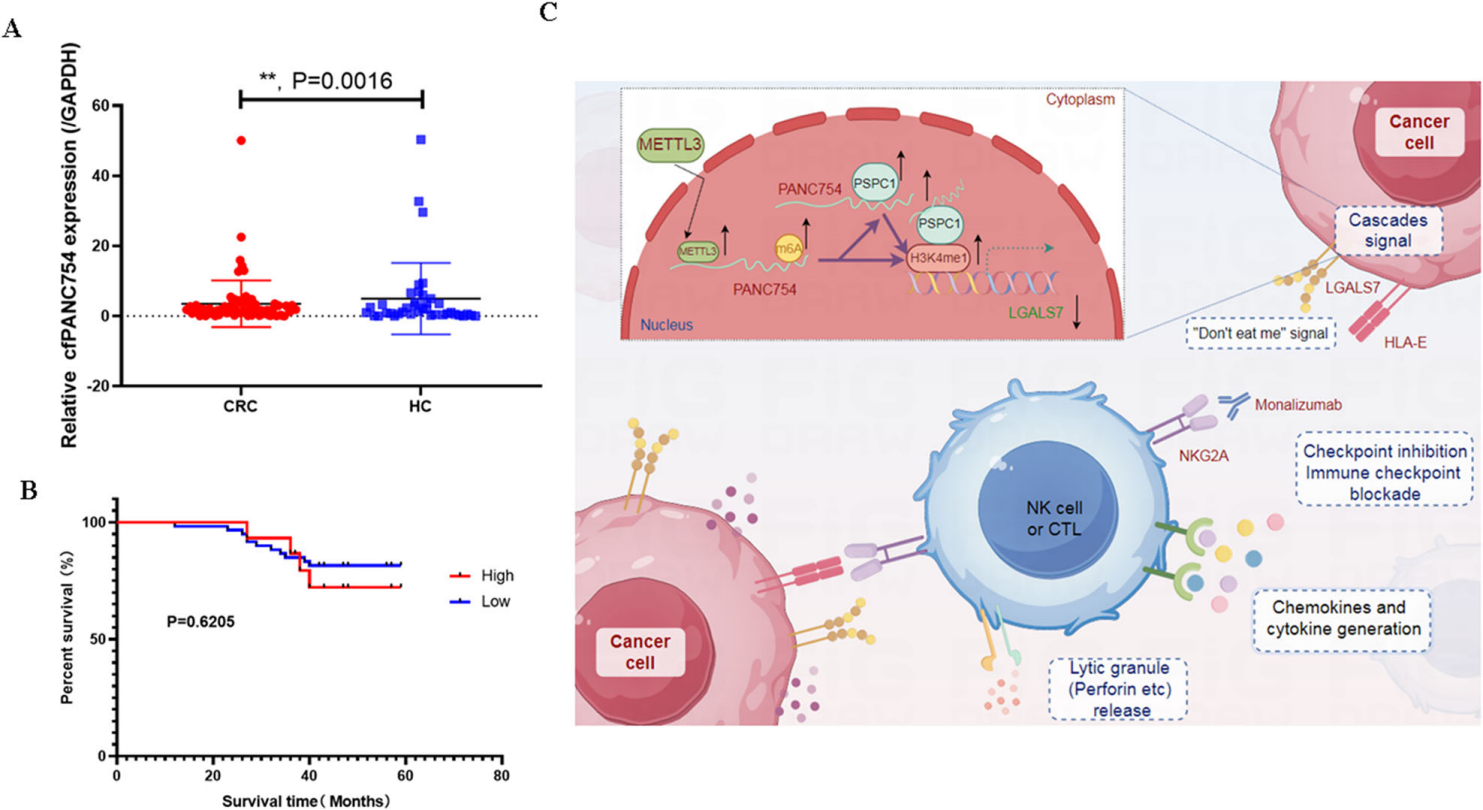
cfPANC754 may serve as a biomarker for the diagnosis of colorectal cancer. **A** The expression level of the cell-free RNA PANC754 in the serum (cfPANC754) from 75 CRC patients and 38 healthy controls was detected by QPCR. CRC, colorectal cancer; HC, healthy control. **B** The survival prediction ability of cfPANC754 by the total survival curve. High, high level of cfPANC754; low, low level of cfPANC754. **C** The graphical abstract of our research indicated that m^6^A/METTL3-dependent nuclear ncRNA PANC754 interacts with its binding protein PSPC1 and chromatin-accessible histone H3K4me1 to form ncRNA/RBP/histone repression complex near LGALS7’s promoter region can enhance immunotherapy capability of ICB anti-NKG2A against colorectal cancer through downregulation of the immune evasive LGALS7 signaling (Created by Figdraw).

As mentioned above, the expression level of PANC754 in CRC tissue is lower than that in adjacent non-cancerous tissue. Therefore, we would like to investigate the expression levels of cell-free PANC754 in the serum (cfPANC754) of CRC patients and healthy individuals. The expression level of cfPANC754 from 75 CRC patients was significantly lower than that from 38 healthy controls, P = 0.0016 (Fig. 8A). The clinical characteristics of 75 CRC cases were shown in Table 1. It is worth mentioning that the differential expression of cfPANC754 was tightly associated with lymph node involvement of CRC metastasis, *P* = 0.035 and UICC stage, P = 0.050, respectively (Table 1). Collectively, cfPANC754 may serve as a biomarker for the diagnosis of CRC. The survival prediction ability of cfPANC754 by the total survival curve, and found that cfPANC754 has no prognostic prediction ability (Fig. 8B).

**Table 1:** Characteristics of the CRC patients included in cfPANC754 detection of this study.

Characteristics	Patient	cfPANC754 level		*P* value
	(*N* = 75)	High	Low	
Age^a^				
≥60	57	12	45	
<60	18	3	15	0.685
Gender				
Male	41	9	32	
Female	34	6	28	0.643
Subtype				
Colon	35	5	30	
Rectal	32	7	25	0.418
Unknown	8	3	5	
Tumor size				
≤4 cm	37	8	29	
>4 cm	32	5	27	0.525
Unknown	6	2	4	
Tumor invasion^b^				
T1 + T2	21	5	16	
T3 + T4	43	8	35	0.627
Tx	11	2	9	
Lymph node^b^				
N0	60	9	51	
≥N1	9	4	5	0.035*
Nx	6	2	4	
Distant metastasis^b^				
M0	61	10	51	
M1	14	5	9	0.103
UICC Stage^c^				
I + II	55	8	47	
III + IV	20	7	13	0.050*
Tumor differentia				
High	9	2	7	
Medium	53	8	45	0.591
Low	5	1	4	
Undefined	8	4	4	

^a^Age stratification was done as previously described. (Colorectal cancer statistics, 2020. CA Cancer J Clin, 2020).

^b^TNM Stages were assessed in accordance with definitions found in the seventh edition of the TNM classification criteria.

^c^UICC Stage: the tumor classification criteria from the Union for International Cancer Control.

Bold values are used to highlight key data with statistical significance.

*P < 0.05.

In a word, our research indicated that m^6^A-dependent nuclear ncRNA PANC754 interacts with its binding protein PSPC1 and chromatin-accessible histone H3K4me1 to form ncRNA/RBP/histone repression complex near promoter region can enhance immunotherapy capability of ICB anti-NKG2A against CRC through downregulation of the immune evasive LGALS7 signaling (Fig. 8C).

## Discussion

Non-coding RNAs were once considered the “junk” of genomes [4]. However, a large body of evidence has demonstrated that many ncRNAs are functional elements instead of “junk RNA” [32, 33]. Functional ncRNAs exist in the tumor and play crucial roles during tumorgenesis [34–37]. In this study, we found a novel, ubiquitously downregulated non-coding RNA transcript, *PANC754*, by scanning the transcript files of the TCGA database across 23 human cancer types. ncRNA *PANC754* is encoded by ENSG00000213754.2, which is a proposed RNA of unknown function (Fig. S8). Functional experiments demonstrate that overexpressed *PANC754* inhibits cell viability, migration, and invasion and induces apoptosis in CRC. The mechanisms are that the nuclear-located PANC754 expression is regulated by m^6^A modification via METTL3 enzyme, which binds with its RBP PSPC1, then interacts with H3K4me1 to form ncRNA/RBP/histone repression complex inhibits immune evasive molecule LGALS7 and leads to suppress CRC progress (Fig. 6G). More interesting, we confirmed that upregulation of the immunotherapeutic ability of Monalizumab by PANC754 combination (Fig. 8B).

Epigenetic modification such as m^6^A can influence the expression of gene transcript [18]. It is reported ncRNA can be widely regulated by m^6^A and METTL3, which generally improve the expression levels of the transcript [19]. Our data also validate that m^6^A modification can control and increase the levels of ncRNA PANC754 in its 1251 A site (Fig. 3A, B). The cellular location of ncRNA can influence the function and its role mode [20]. We found PANC754 is in the cell nucleus, so we explored its RBPs through RNA pulldown and LS-MS/MS and found a TF, PSPC1 with an RRM to bind with PANC754 (Fig. 4). The RNA/protein complex accumulates at the promoter region of genes can enhance or suppress the expression of genes [7]. This epigenetic regulation mode is intrinsic, complicate, and effective. In this article, we find that ncRNA PANC754, PSPC1 protein, and chromatin-accessible histone H3K4me1 protein can form an ncRNA/RBP/histone repression complex to suppress gene expression for the first time.

*LGALS7* gene is a lectin that belongs to the Galectin family of proteins [38]. Galectins are known for their ability to bind β-galactoside-containing carbohydrates, which are present on the surface of many cells [39]. LGALS7 plays a crucial role in immune cell function, inflammation, cell adhesion, migration, proliferation, and apoptosis; It has been implicated in the development and progression of various diseases, including CRC and other cancers [6, 38, 39]. In the context of cancer, LGALS7 has been shown to modulate tumor cell behavior, potentially promoting or inhibiting tumor growth and metastasis, depending on the tumor type and microenvironment [6, 39]. Additionally, it may serve as a biomarker for certain cancers through binding to the immune cell surface and could be a target for therapeutic intervention [39]. Furthermore, *LGALS7* gene serves as a flag of “Don’t eat me” in fostering innate immune evasive programs [6, 22]. Hence, PANC754 with its RBP/histone repression complex suppresses LGALS7 level can improve the recognition and phagocytosis of the tumor cells by NKs and CTLs.

Immune therapeutics especially immune checkpoint inhibitors (ICIs) are a promising strategy for tumor patients including CRC [40]. However, the efficacy of ICIs can be quite variable among patients, depend on the composition and state of the tumor microenvironment, the level of immune cell infiltration, etc [41–43]. The use of ICIs in combination with other treatments, such as RNA molecular therapy can enhance their effectiveness [43].

NKG2A is a major receptor of the cell surface of NKs or CTLs, and NKG2A/CD94 heterodimer forms an inhibitory receptor [26, 27]. Disruption of the NKG2A:HLA-E immune checkpoint axis to enhance NK or CTL activation against cancer is a hotspot of immunotherapy [6, 22, 27]. Monalizumab monoclonal antibody is an anti-NKG2A ICI that targets the NKG2A gene [28, 44–46]. In this study, we found that ncRNA PANC754 can downregulate the level of LGALS7 on the CRC cell surface, so we tried to combine the overexpression of PANC754 with Monalizumab in a CRC and PBMCs co-culture system, in order to evaluate whether PANC754 increases the effect of Monalizumab. As was expected, ncRNA PANC754 increases the immune therapeutics ability of this anti-NKG2A ICI by improving the release of Perforin to CRC cells (Fig. 7B), the solid evidence is that the increasing concentration of LDH after PANC754 and Monalizumab combination therapy (Fig. 7D). Although the release of GZMB enzyme is not notable, maybe due to our using of the mixed immune cells, PBMCs, which the difference of immune cell types and CRC cell types can confer different results.

Although our research emphasized that PANC754 downregulates the level of the “Don’t eat me” signal LGALS7 to improve the immune efficiency of ICB and induce NK or CTL cells to release Perforin and cytokines to kill tumor cells. The shortage is a deep study of LGALS7 function. The clinical translational study of PANC754 and Monalizumab combination is another future focus. We will further focus on these inadequacies to study in the near future.

## Supplementary information

Supplementary Methods and Results

Supplementary Tables

Supplemental Material file about WB

## Data Availability

The data of the current study are available from the corresponding author upon reasonable request.
