# Supplementary material for: M6A-METTL3-dependent nuclear PANC754/PSPC1/H3K4me1 repression complex regulate immune evasive LGALS7 signal to enhance immunotherapy against colorectal cancer
Source: Cell Death Dis. 2025 Jul 9;16(1):506. doi: 10.1038/s41419-025-07820-9 (PMC12241656; doi:10.1038/s41419-025-07820-9)
Supplement: Supplementary file 1 — Supplementary Methods and Results [file 41419_2025_7820_MOESM1_ESM.docx]

**M^6^A-METTL3-dependent nuclear PANC754/PSPC1/H3K4me1 repression complex regulate immune evasive LGALS7 signal to enhance immunotherapy against colorectal cancer**

**Supplementary Files**

**Supplementary Methods and materials**

**Detection of cell growth viability by CCK-8 assay**

Cells at a concentration of 5 x10^3^ per well were seeded in 96-well plates and incubated for 24 h. At 24, 48, and 72 h after transfection, cell growth and viability was measured with a Cell Counting Kit-8 (Beyotime, China), following manufacturer’s instructions. Absorbance at 450 nm (OD_450_) was then recorded using Multiskan Sky Reader (Thermo Electrom, USA).

**Detection of apoptosis by flow cytometry**

Cells were trypsinized and washed twice in ice-cold phosphate buffered saline (PBS). A total of 5 ×10^5^ cells were resuspended in 500 µL of binding buffer (KeyGen). 5 µL of Annexin V-FITC (KeyGen) or CFDA-SE (Beyotime) was then added to the cell suspension which was counterstained with 5 µL of propidium iodide (PI). The mixture was incubated at room temperature for 10 min in the dark followed by apoptosis analysis using the FlowSight Flow Cytometer (Merck, Germany).

**Determination of cell invasion by transwell chamber assay**

The cell invasion assay was performed by using an invasion chamber (Chemicon, USA), which is a 24-well tissue culture plate with 12-cell culture inserts. Cell suspensions (0.5×10^5^ cells/mL) transfected by pcDNA3.1-PANC754, untransfected control, and pcDNA3.1 were added to the interior of the inserts with 200 µL serum-free media while 500 µL of media containing 10% FBS was added to the lower chamber as a migration stimulus. The transwell chambers were incubated in a tissue culture incubator for 24 h. The invasive cells on lower surface of the membrane were stained by crystal violet for 10 min and images taken at 40X number of high magnification fields (HMF) of the microscope.

**Nuclear-cytoplasmic fractionation experiment**

Cells were lysed inNE-PER extraction reagent (Pierce, USA) and processed according to the manufacturer's instructions. The lysed cells were centrifuged at high speed to pellet the nuclei and separate them from the cytoplasmic fraction. The cytoplasmic supernatant was then carefully removed and stored separately. The mRNA expression levels of *U6*, *GAPDH*, and *PANC754* were determined by RT-PCR in the cytoplasmic and nuclear fraction.

**Molecular docking experiment**

Rigid protein–protein docking (ZDOCK) was performed between H3K4me1 and PSPC1 to study the relationships. The PDB format of the protein structural domain was downloaded from the Protein Data Bank PDB database (http://www.rcsb.org/). The ZDOCK module was run to identify the docking sites and calculate the ZDOCK scores. Protein interaction prediction portal (http://genemania.org/) was used to predict the protein-protein interplay.

**Lentivirus infection of CRC cell line**

Seed CRC cells into cell culture flasks and add an appropriate amount of culture medium and fetal bovine serum. Incubate the cells at 37°C and 5% CO_2_ in a cell incubator. When the cells grow to 70% to 80% confluence, digest them with trypsin and passage the cells to new cell culture flasks. Add lentivirus 1x10^8^ TU/mL of PANC754, METTL3 (Genecreate lnc.) to the cell culture flask and gently shake the flask to fully infect the cells. Incubate the cells for an additional 48 h at 37°C and 5% CO_2_ in a cell incubator. After 48 h, observe the infection status of the cells using an inverted fluorescence microscope.

**Detection of Perforin, Granzyme B by ELISA**

150 µL of the cell culture supernatant was carefully added to the U-shaped wells of the ELISA (enzyme-linked immunosorbent assay) plates from Perforin and Granzyme B ELISA kits (Saipeisen Bio, China) according to the reagents' instruction. The optical density (OD) at 450 nm was measured using a high-sensitivity microplate reader from BioTek, USA, to quantify the levels of Perforin and Granzyme B present in the culture supernatants.

**Supplementary Results**

**Figure S1-1. PANC754 markedly inhibited cell growth and metastasis of CRC cell lines. (A)** The transfection effiency of overexpression of PANC754 was determined by the fluorescence microscope. **(B)** The expression levels of PANC754 were validated in the normal [intestinal epithelial cell](javascript:;) (NCM460) and various types of CRC cell lines. Each experiment was repeated at least three times. **(C)** Cell apoptosis of SW480 cells detection by flow cytometry. PI, Propidium Iodide. **(D)** The proliferative curve of DLD1 cell line with overexpression of PANC754 by CCK-8 assay. Control, untransfected SW480; pcDNA3.1, empty pcDNA3.1 plasmid transfected; PANC754, overexpression plasmid of PANC754 transfected; ***, P<0.001.


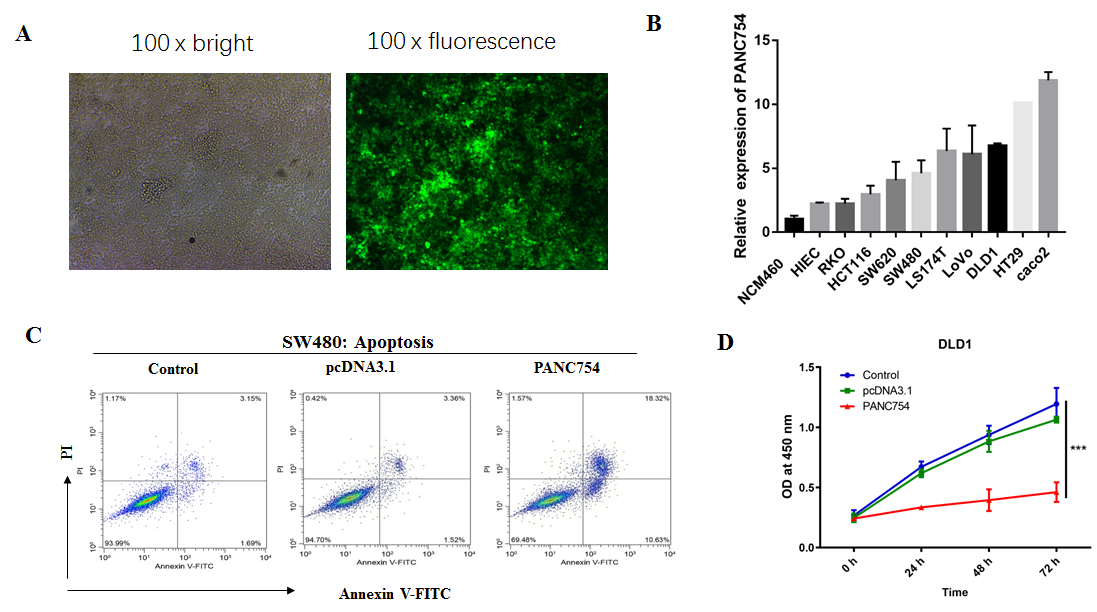


**Figure S1-2. PANC754 markedly inhibited cell growth and metastasis of CRC cell lines. (A)** Determination of cell migration in SW480 cell line by wound healing repair assay and its statistic histograms in 24 h **(B)** or 48 h **(C)**. ns, no significance; ***, P<0.001.(**D,E**) EMT markers were detected by WB and their statistic histograms in SW480 cell line. *, P<0.05. **, P<0.01. ns, no significance. Each experiment was repeated at least three times.


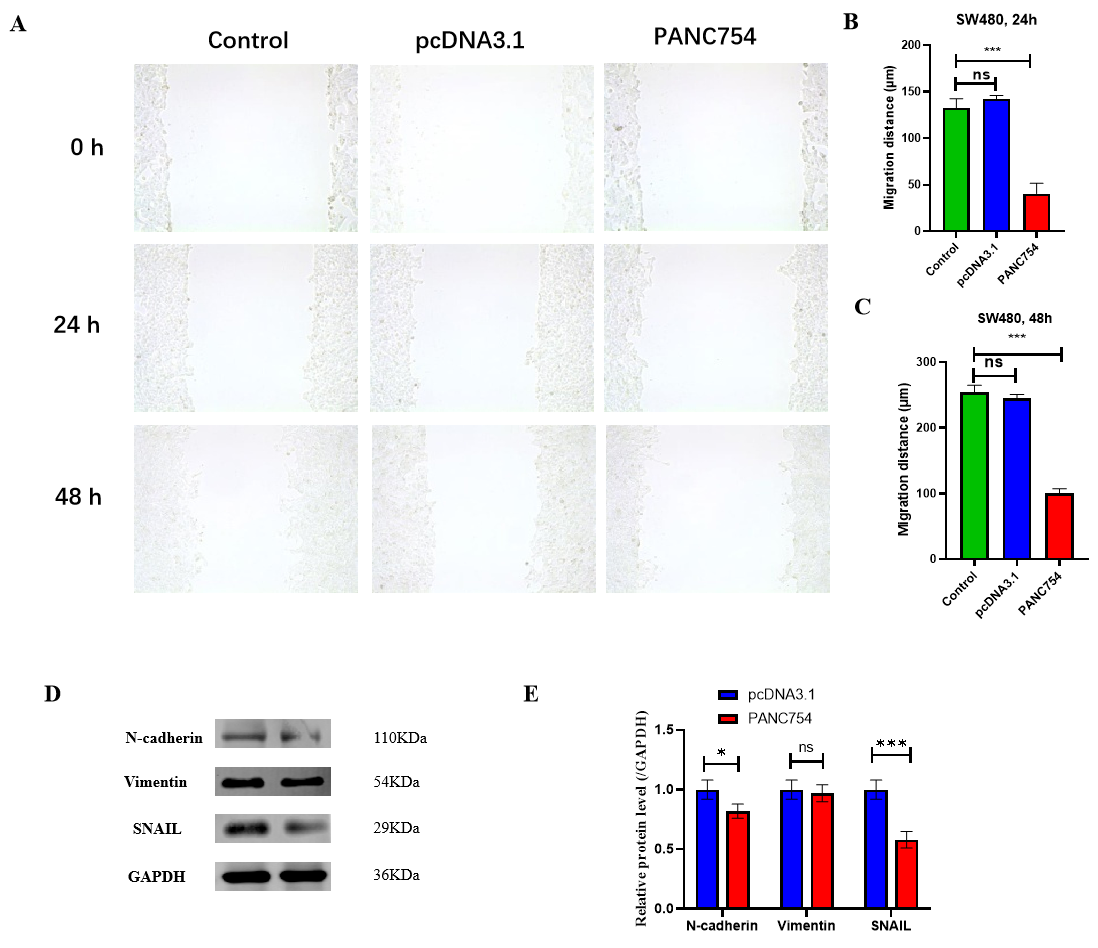


**Figure S2. PANC754 significantly suppressed cell growth and metastasis in CDX model. (A)** The presentation of the CDX model and tumor in three different treatment groups (n = 5, respectively). WT, lentivirus uninfected SW480; Empty, empty lentivirus infected; PANC754, overexpression PANC754 lentivirus infected. **(B)** The weight of CDX models in three treated CDX groups. Empty vector, empty lentivirusvector infected; OE-PANC754, overexpression PANC754 lentivirus vector infected.ns, no significance.


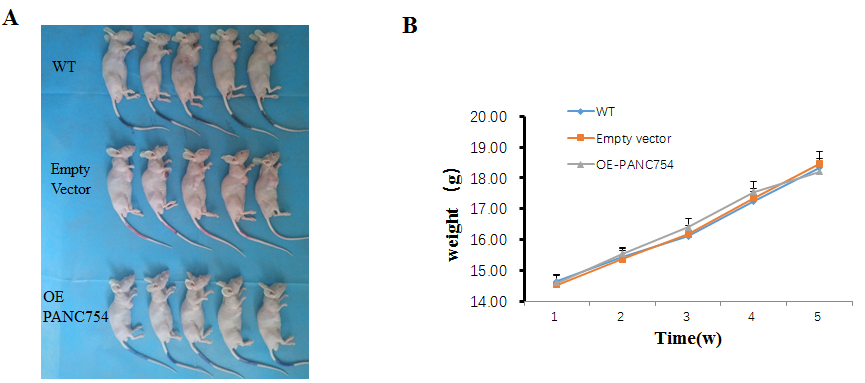


**Figure S3. *PANC754* was regulated by m^6^A modification with methyltransferase METTL3. (A)** Prediction of m^6^A site, motif and score of *PANC754* in the SRAMP online website. High combined score imply high confidence. **(B)** The m^6^A level of *PANC754* gene was detected by meRIP-PCR. Input as a positive control.


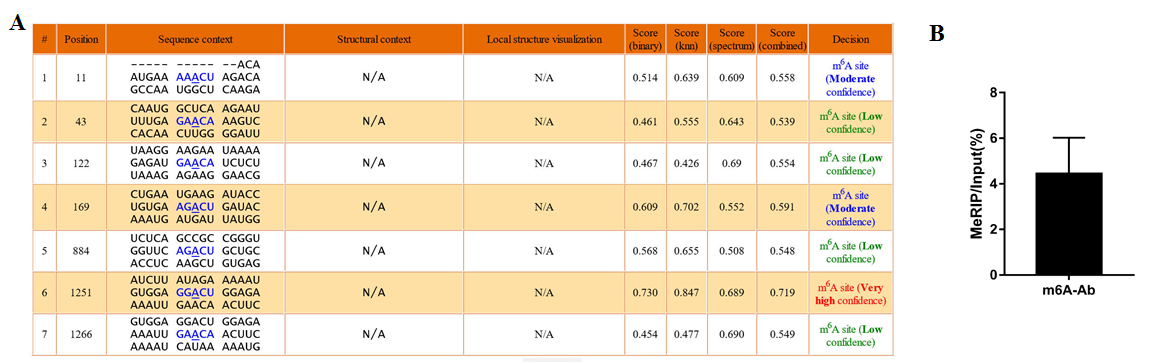


**Figure S4. PANC754 was the nuclear-located and bound with its RBP PSPC1. (A,B)** The representational mass spectrum peak image of both sense (A) and antisense RNA transcripts (B) of *PANC754* by LS-MS/MS detection. **(C)** The structure diagram of PSPC1 protein. **(D)** The 3D crystal structure of PSPC1 protein derived from PDBs database.


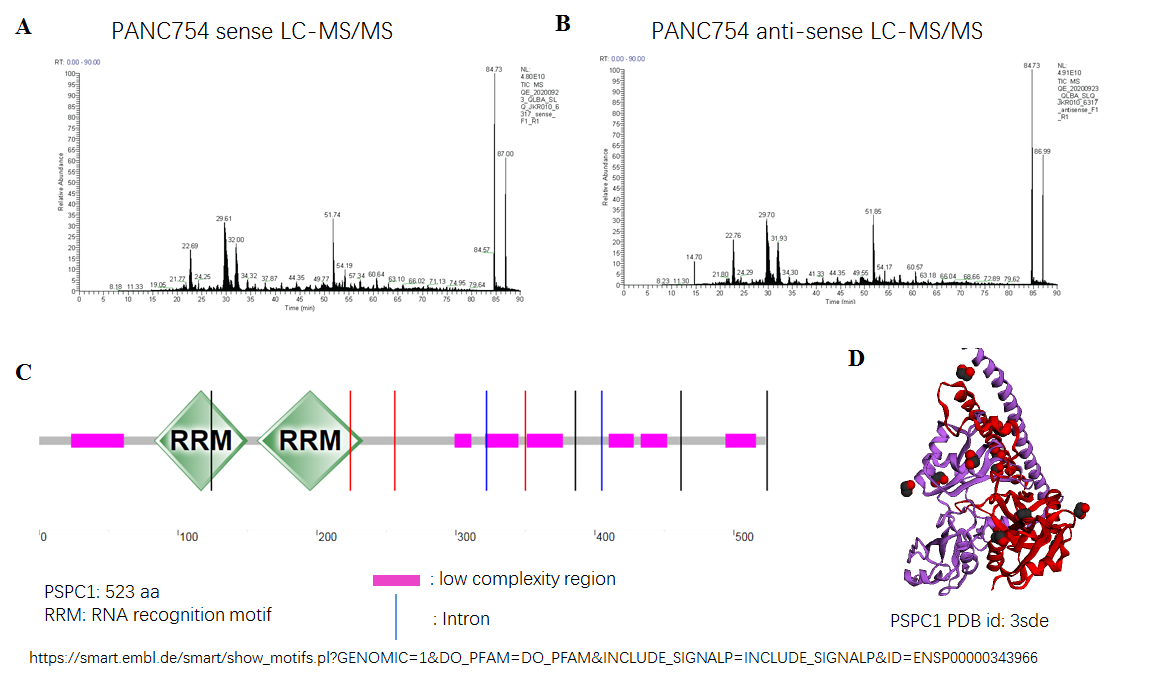


**Figure S5. PANC754 suppresses CRC progress via inhibiting immune evasive molecule LGALS7. (A)** The knockdown effect of two pairs of shPANC754 was validated by qRT-PCR in SW480 cells. shControl, scrambled shRNA plasmid transfected; shPANC754-1, shRNA-1 plasmid against PANC754 transfected. shPANC754-2, shRNA-2 plasmid against PANC754 transfected. **(B)** The three pairs of shRNA sequence against *PANC754* gene were listed.

**
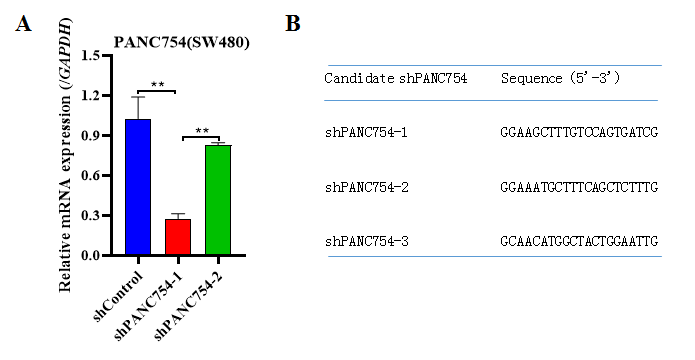
**

**Figure S6-1. PSPC1 and H3K4me1 interaction regulates LGALS7 expression. (A)** The gene ontology (GO) analysis from RNA-Seq data between normal control and PANC754 overexpression groups. CC, cellular component; MF, molecular function; BP, biological process. NC, normal control; PANC754, PANC754 overexpression. **(B)** The bubble chart of GO analysis indicated the nuclear intersection of PANC754.


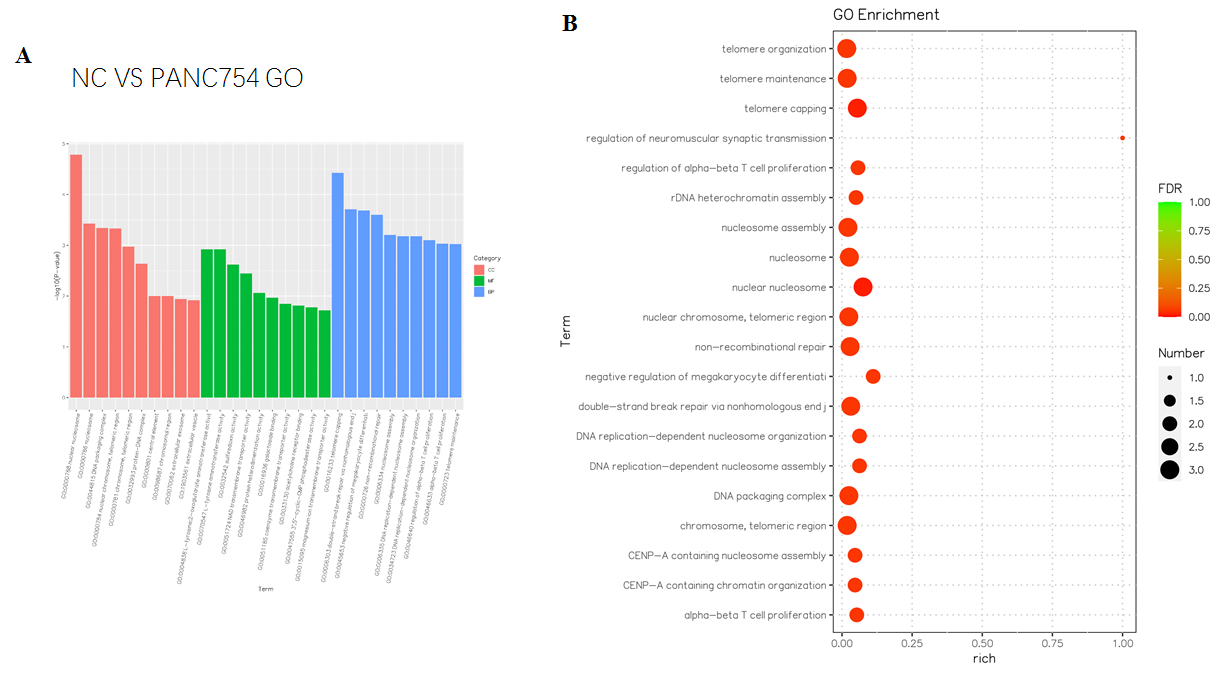


**Figure S6-2. PSPC1 and H3K4me1 interaction regulates LGALS7 expression. (A)** The Kyoto Encyclopedia of Genes and Genomes (KEGG) analysis diagram from RNA-Seq data between normal control and PANC754 overexpression groups. CC, cellular component; MF, molecular function; BP, biological process. NC, normal control; PANC754, PANC754 overexpression. **(B)** The bubble chart of KEGG pathway enrichment analysis.


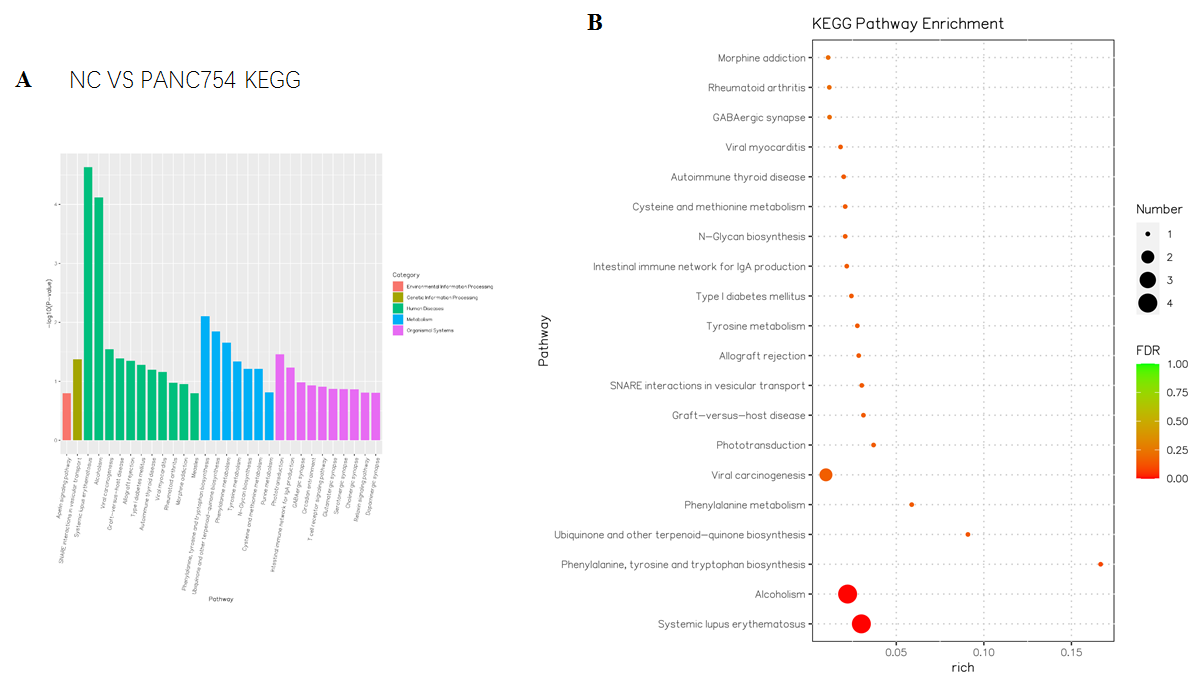


**Figure S6-3. PSPC1 and H3K4me1 interaction regulates LGALS7 expression. (A)** The University of California Santa Cruz (UCSC) genome browser analysis of the 5’UTR (especially promoter region) of *LGASL7* gene showed that H3K4me1 and H3K27ac were enriched. **(B)** The interaction among PSPC1, H3-4, and METTL3 in Protein interaction prediction portal. **(C)** The histone H3K4ac protein level after PANC754 overexpression in SW480 cells was detected WB and their statistic histograms **(D).** Histone H3 as a control. *, P<0.05. Each experiment was repeated at least three times.


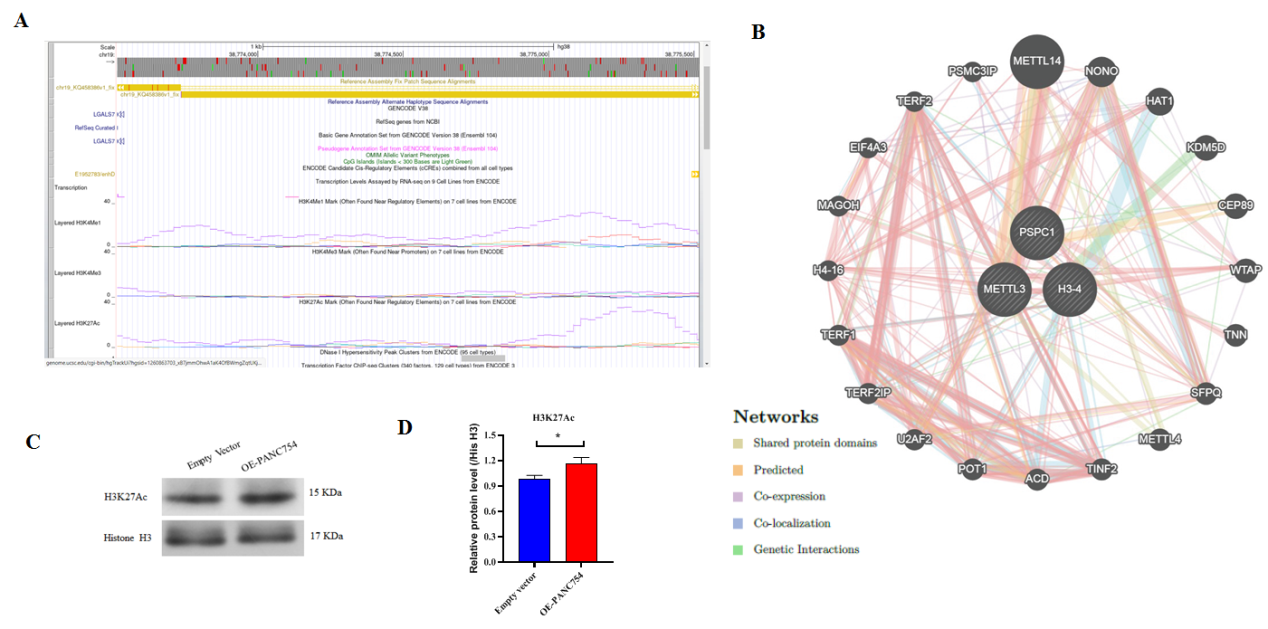


**Figure S7-1. Up-regulation of the immunotherapeutic ability of monalizumab by PANC754 combination. (A)** The representative apoptosis figure of HCT116 cells detected by FCM. NC, empty plasmid. **(B)** The mRNA level of *PANC754* gene in PBMCs was determined by qRT-PCR. PF, Perforin; CRC, HCT116 cells; PBMC, peripheral blood mononuclear cells; OE-PANC754, transfected with PANC754 overexpression plasmid. *, P<0.05; **, P<0.01;***, P<0.001; ****, P<0.0001; ns, no significance. **(C)** The mRNA level of *NAKG2D* gene in PBMCs (Effect cells) was determined by qRT-PCR. **(D)** The mRNA level of *NAKG2A* gene in HCT116 cells (Target cells) was determined by qRT-PCR. **(E)** The mRNA level of *NAKG2D* gene in HCT116 cells determined by qRT-PCR. **(F)** The mRNA level of *LGALS7* gene in PBMCs from co-culture system was determined by qRT-PCR. Each experiment was repeated at least three times.


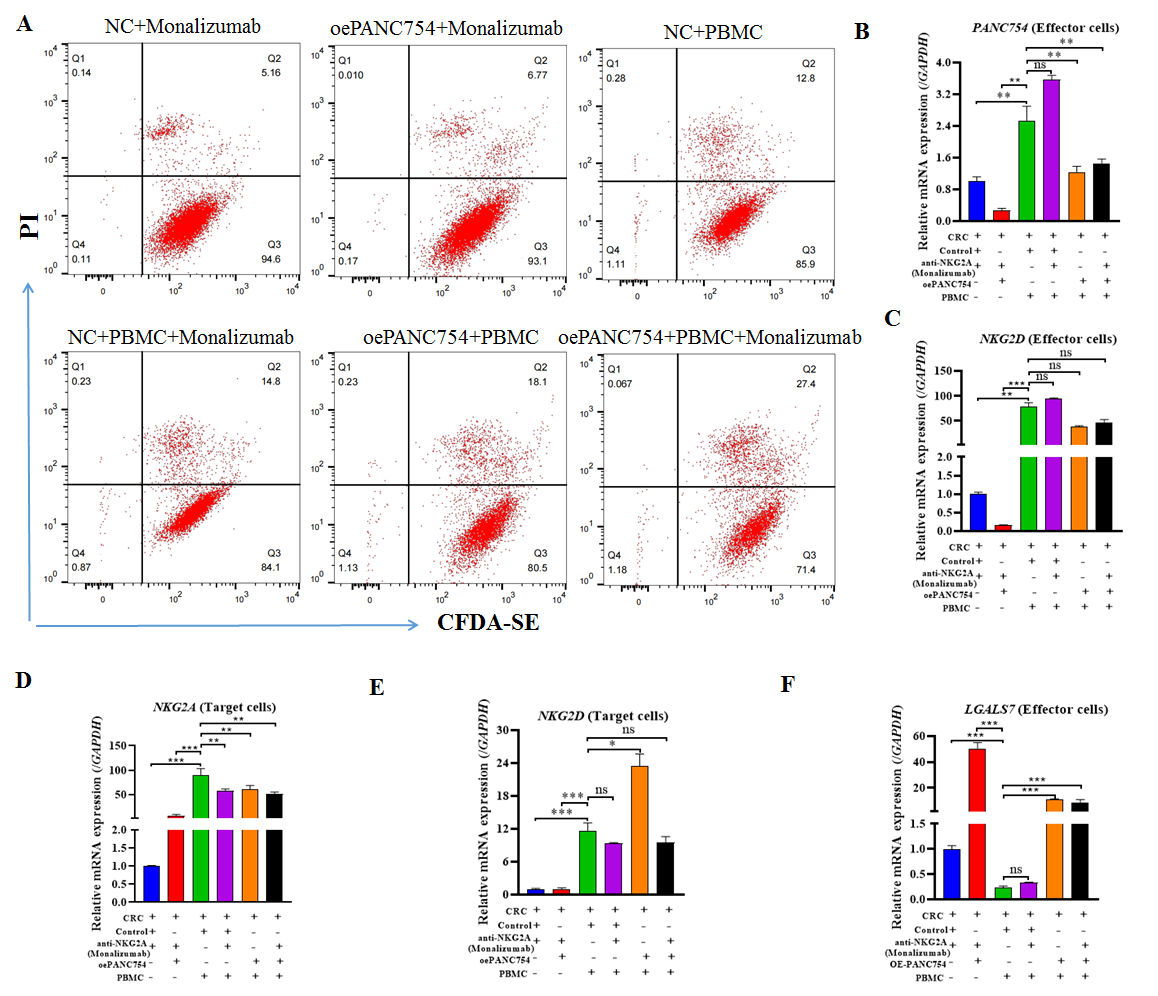


**Figure S7-2. Up-regulation of the immunotherapeutic ability of monalizumab by PANC754 combination. (A)** The representative ICC staining figure of E-cadherin protein in HCT116 cells. **(B)** The representative ICC staining figure of MMP9 protein in HCT116 cells from co-culture system. i) group, NC+Monalizumab: CRC+Control+Monalizumab; ii) group, NC+PBMC: CRC+Control+PBMC; iii) group, NC+PBMC+Monalizumab: CRC+Control+PBMC+Monalizumab; iv) group, oePANC754+Monalizumab: CRC+OE-PANC754+Monalizumab; v) group, oePANC754+PBMC: CRC+OE-PANC754+PBMC; vi) group, oePANC754+PBMC+Monalizumab: CRC+OE-PANC754+PBMC+Monalizumab. The individual use of monalizumab: refered to i) group; The individual use of PANC754: refered to v) group; The combination therapy of monalizumab and PANC754: refered to vi) group.


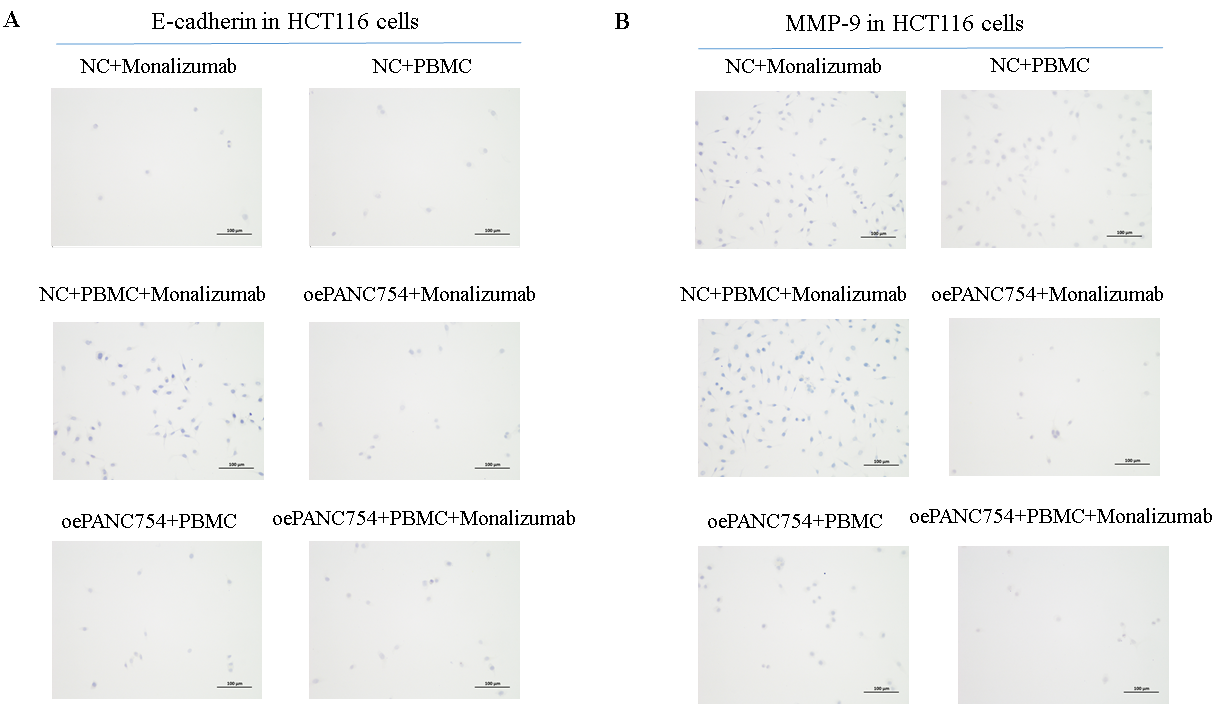


**Figure S8. The sequence difference between KPNA4P1 and PANC754 by BLASTN (A)** By exploring Ensemble database, we found ncRNA ENSG00000213754.2 was be nominated as KPNA4P1 in July 2023, but named later than our patent application. **(B)** Through serials of study, we found the ncRNA ENSG00000213754.2 played a pan-cancer suppression role, so we nominated it as PANC754. However, PANC754 is not similar with KPNA4P1 through BLASTN.

**A.**


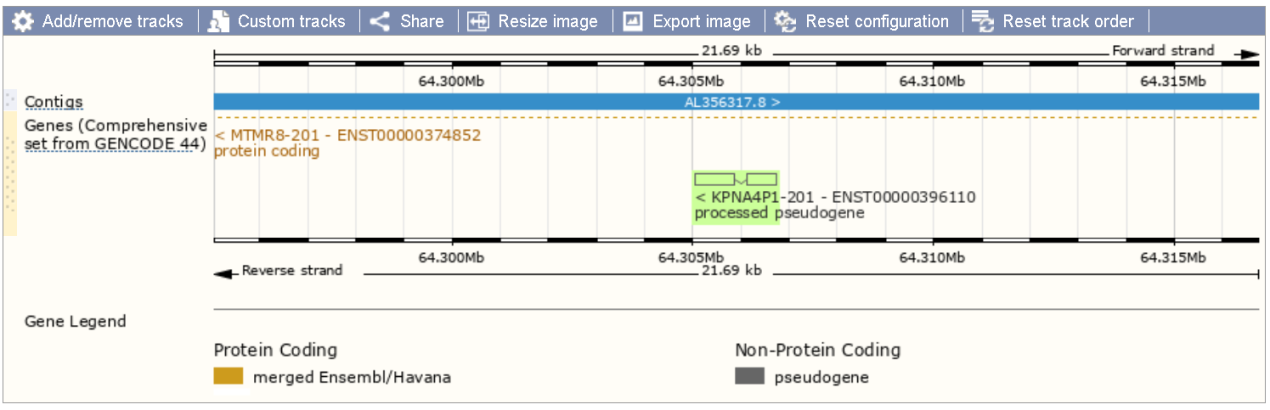


**B.**

Query: None Query ID: lcl|Query_2101229 Length: 2057 (*KPNA4P1*)

>

Sequence ID: Query_2101231 Length: 1435 (*PANC754*)

Range 1: 1 to 1435

Query 1 GCTGGGGTTGAGGAAGGGAGTTGTACAGGGAGAGGAGAAGGCCTCAGCTGAGGCAGTTGA 60

Query 61 GGAGGAGGAAGGAAGCAGTGGCACAGCCGGCATAGCAGCACAGGCAGCCTGGTTCCTGGG 120

Query 121 AGTGTCGGTGTGGCATGGTGGTTGGGGCAGATCCTGTGGGGCCTGGGTAGGGGAAGGAGT 180

Query 181 CATCAGCTCGTTCATAGTGGACAATGAAAAACTAGACAGCCAATGGCTCAAGAATTTTGA 240

||||||||||||||||||||||||||||||||||||||||

Sbjct 1 --------------------ACAATGAAAAACTAGACAGCCAATGGCTCAAGAATTTTGA 40

Query 241 GAACAAAGTCCACAACTTGGGGATTATGAGAAGACAGCAAAATGAAGTTGTAGTTGAATT 300

||||||||||||||||||||||||||||||||||||||||||||||||||||||||||||

Sbjct 41 GAACAAAGTCCACAACTTGGGGATTATGAGAAGACAGCAAAATGAAGTTGTAGTTGAATT 100

Query 301 AAGGAAGAATAAAAGAGATGAACATCTCTTAAAGAGAAGGAACGTACTGAATGAAGATAC 360

||||||||||||||||||||||||||||||||||||||||||||||||||||||||||||

Sbjct 101 AAGGAAGAATAAAAGAGATGAACATCTCTTAAAGAGAAGGAACGTACTGAATGAAGATAC 160

Query 361 CTGTGAAGACTGATACAAATGATGATTATGGAGTGTAAAATACCTCTCTAGTAGCTATTA 420

||||||||||||||||||||||||||||||||||||||||||||||||||||||||||||

Sbjct 161 CTGTGAAGACTGATACAAATGATGATTATGGAGTGTAAAATACCTCTCTAGTAGCTATTA 220

Query 421 TTCAAAATGCTTCAAGTGATAACTAAGAAATTCAATTAAATGCAGTTCGGGCTGCTAGGA 480

||||||||||||||||||||||||||||||||||||||||||||||||||||||||||||

Sbjct 221 TTCAAAATGCTTCAAGTGATAACTAAGAAATTCAATTAAATGCAGTTCGGGCTGCTAGGA 280

Query 481 AGCTTTGTCCAGTGATCGAAGTCTACCAATTTATAAAAATAAAATCTGGAATACTACCTG 540

||||||||||||||||||||||||||||||||||||||||||||||||||||||||||||

Sbjct 281 AGCTTTGTCCAGTGATCGAAGTCTACCAATTTATAAAAATAAAATCTGGAATACTACCTG 340

Query 541 TTCTAGTCTATTGTCTTGAAAGAGATGACAATGCTTTACAGTTAGAAGCTGCATGGGCTT 600

||||||||||||||||||||||||||||||||||||||||||||||||||||||||||||

Sbjct 341 TTCTAGTCTATTGTCTTGAAAGAGATGACAATGCTTTACAGTTAGAAGCTGCATGGGCTT 400

Query 601 TGACAAACACTGCATCTAGAACCTCTGAACAAGCTCAAACAGTAGTTCAGTCCAATGCTG 660

||||||||||||||||||||||||||||||||||||||||||||||||||||||||||||

Sbjct 401 TGACAAACACTGCATCTAGAACCTCTGAACAAGCTCAAACAGTAGTTCAGTCCAATGCTG 460

Query 661 TGCCACATTTCCTGAGGCTCCTCCATTCACTTCATCAGAATGTTTGTGAGCAAGCAGTGT 720

||||||||||||||||||||||||||||||||||||||||||||||||||||||||||||

Sbjct 461 TGCCACATTTCCTGAGGCTCCTCCATTCACTTCATCAGAATGTTTGTGAGCAAGCAGTGT 520

Query 721 GGGCATTGAGAACTATCATAAGTGATGGTCCTCCCGATGTAGAGATTATGTCATAAGTCT 780

||||||||||||||||||||||||||||||||||||||||||||||||||||||||||||

Sbjct 521 GGGCATTGAGAACTATCATAAGTGATGGTCCTCCCGATGTAGAGATTATGTCATAAGTCT 580

Query 781 TGGAGTTGTGAAACCTTTATTTTACTTCAAAACATTTTTTTTTTCTGTGATAGGGGCTCA 840

||||||||||||||||||||||||||||||||

Sbjct 581 TGGAGTTGTGAAACCTTTATTTTACTTCAAAA---------------------------- 612

Query 841 CCCAGGCTGGAATGCAGTGGCGCAAACACAGCTAACTGCACCCTCAACCTCTGGAGCTCA 900

Sbjct ------------------------------------------------------------

Query 901 AGAAATCATCCTGCCTTAGCCTCCCGAGTAGCTGGAACCACAAGTGCGTGCCACCACACC 960

Sbjct ------------------------------------------------------------

Query 961 CAGCTAAATGTTTAAATTGTTTGTAGAGACAGGGTCTCACCATGTTGCCTAGGCTGGTCT 1020

Sbjct ------------------------------------------------------------

Query 1021 CTAACTCCTGGACTCAAGAATCCTCCTGCCTCAGCCTCCCAAAGTGCTAGCATTCCAAGT 1080

|||||||||||||||||

Sbjct 613 -------------------------------------------GTGCTAGCATTCCAAGT 629

Query 1081 GTGAGCCACCATGCCCAGCCTGGGTTATGGCCAATTTATGTCACCACAAAGAGCCACCAC 1140

||||||||||||||||||||||||||||||||||||||||||||||||||||||||||||

Sbjct 630 GTGAGCCACCATGCCCAGCCTGGGTTATGGCCAATTTATGTCACCACAAAGAGCCACCAC 689

Query 1141 CACCAATGGAAACCATACAGGAAATGCTTTCAGCTCTTTGTGTTCTAATTCATCATACAT 1200

||||||||||||||||||||||||||||||||||||||||||||||||||||||||||||

Sbjct 690 CACCAATGGAAACCATACAGGAAATGCTTTCAGCTCTTTGTGTTCTAATTCATCATACAT 749

Query 1201 TTGTAAATATATTAGTAGATACAGACTGGGCCCTCTCTTACCTTACTGGTGCTGGCAATG 1260

||||||||||||||||||||||||||||||||||||||||||||||||||||||||||||

Sbjct 750 TTGTAAATATATTAGTAGATACAGACTGGGCCCTCTCTTACCTTACTGGTGCTGGCAATG 809

Query 1261 AACAAATACAGATGGTAGTAGACTCTGGAATAGTTCCTCATTTGGTTCCTCTTCTCAGCC 1320

||||||||||||||||||||||||||||||||||||||||||||||||||||||||||||

Sbjct 810 AACAAATACAGATGGTAGTAGACTCTGGAATAGTTCCTCATTTGGTTCCTCTTCTCAGCC 869

Query 1321 GCCGGGTGGTTCAGACTGCTGCACCTCAAGCTGTGAGCAACATGGCTACTGGAATTGAAG 1380

||||||||||||||||||||||||||||||||||||||||||||||||||||||||||||

Sbjct 870 GCCGGGTGGTTCAGACTGCTGCACCTCAAGCTGTGAGCAACATGGCTACTGGAATTGAAG 929

Query 1381 AGCAAACAGAAGTAGTTGTGAATTATGATGCTCTTTCATACTTGTGGGCAATCCTGACAC 1440

||||||||||||||||||||||||||||||||||||||||||||||||||||||||||||

Sbjct 930 AGCAAACAGAAGTAGTTGTGAATTATGATGCTCTTTCATACTTGTGGGCAATCCTGACAC 989

Query 1441 ATCCCAAAGAGAAAATTAATAAAGAGGCAGTATGGTTTCTCTGTAACATCACTGCAGGTA 1500

||||||||||||||||||||||||||||||||||||||||||||||||||||||||||||

Sbjct 990 ATCCCAAAGAGAAAATTAATAAAGAGGCAGTATGGTTTCTCTGTAACATCACTGCAGGTA 1049

Query 1501 ATCAGCTGCAGATACAGGCAGTAATAGATGCCAATATTGTACCAAGAATAATAAACCTTT 1560

||||||||||||||||||||||||||||||||||||||||||||||||||||||||||||

Sbjct 1050 ATCAGCTGCAGATACAGGCAGTAATAGATGCCAATATTGTACCAAGAATAATAAACCTTT 1109

Query 1561 TGGATAAGGGGGATATTGGCACTCAGAAGCTGACTGTAAAAGATGCACAAGTTGTACAAG 1620

||||||||||||||||||||||||||||||||||||||||||||||||||||||||||||

Sbjct 1110 TGGATAAGGGGGATATTGGCACTCAGAAGCTGACTGTAAAAGATGCACAAGTTGTACAAG 1169

Query 1621 TAGTACTGGATGAATTAATATATTAAAAATGGCTAAAAATGAGGCAGAATCCATAGCCAA 1680

||||||||||||||||||||||||||||||||||||||||||||||||||||||||||||

Sbjct 1170 TAGTACTGGATGAATTAATATATTAAAAATGGCTAAAAATGAGGCAGAATCCATAGCCAA 1229

Query 1681 TCTTATAGAAAAATGTGGAGGACTGGAGAAAATTGAACAACTTCAAAATCATAAAAATGG 1740

||||||||||||||||||||||||||||||| ||||||||||||||||||||||||||||

Sbjct 1230 TCTTATAGAAAAATGTGGAGGACTGGAGAAA-TTGAACAACTTCAAAATCATAAAAATGG 1288

Query 1741 AGACATCTACAAATTAGCCTATGAGATGATTGCTCAGTTCTCTTCAAATGATATTGATGA 1800

||||||||||||||||||||||||||||||||||||||||||||||||||||||||||||

Sbjct 1289 AGACATCTACAAATTAGCCTATGAGATGATTGCTCAGTTCTCTTCAAATGATATTGATGA 1348

Query 1801 AGATCTTAGCCTTGATCCAGAGGCAATTCAAGGTGGAACATTTGGTTTCAATTTATCTGC 1860

||||||||||||||||||||||||||||||||||||||||||||||||||||||||||||

Sbjct 1349 AGATCTTAGCCTTGATCCAGAGGCAATTCAAGGTGGAACATTTGGTTTCAATTTATCTGC 1408

Query 1861 CAGTATACCAATAGAACAGTTCCAGTTTTAGAACAGTATTGTGGAAGTTAGGTACAATTT 1920

|||||||||||||||||||||||||||

Sbjct 1409 CAGTATACCAATAGAACAGTTCCAGTT--------------------------------- 1435

Query 1921 ATATACATACATACATACGTATATATGTATATACATATATATGTATATACATATATATAT 1980

Query 1981 GTTTATACATATATGTGTGTATATGTGTATATATATATGTATGTGTATATATATATATAT 2040

Query 2041 ATATATATATATATATA 2057
