# Supplementary material for: M6A-METTL3-dependent nuclear PANC754/PSPC1/H3K4me1 repression complex regulate immune evasive LGALS7 signal to enhance immunotherapy against colorectal cancer
Source: Cell Death Dis. 2025 Jul 9;16(1):506. doi: 10.1038/s41419-025-07820-9 (PMC12241656; doi:10.1038/s41419-025-07820-9)
Supplement: Supplementary file 2 — Supplementary Tables [file 41419_2025_7820_MOESM2_ESM.docx]

**M^6^A-METTL3-dependent nuclear PANC754/PSPC1/H3K4me1 repression complex regulate immune evasive LGALS7 signal to enhance immunotherapy against colorectal cancer**

**Supplementary Tables**

**Table S1 Characteristics of the CRC patients included in PANC754 detection of our study**

| **Characteristics** | **Patient Distribution** |
| --- | --- |
|  | **(N = 26)** |
| **Age ^a^** |  |
| ≥60 | 19 |
| <60 | 7 |
| **Gender** |  |
| Male | 12 |
| Female | 14 |
| **Subtype** |  |
| Colon | 15 |
| Rectal | 11 |
| **Tumor size** |  |
| ≤4 cm | 17 |
| >4 cm | 9 |
| **UICC Stage** |  |
| I+ II | 18 |
| III+ IV | 8 |
| **Tumor invasion depth ^b^** |  |
| T1+T2 | 15 |
| T3+T4 | 8 |
| Tx | 3 |
| **Lymph node involvement^b^** |  |
| N0 | 11 |
| **≥**N1 | 9 |
| Nx | 6 |
| **Distant metastasis ^b^** |  |
| M0 | 22 |
| M1 | 4 |
| **Tumor differentia depth ^b^** |  |
| High | 4 |
| Medium | 16 |
| Low | 4 |
| Undefined | 2 |

^a^Age stratification was done as previously described. (Colorectal cancer statistics, 2020. CA Cancer J Clin, 2020)

^b^TNM Stages were assessed in accordance with definitions found in the seventh edition of the TNM classification criteria.

**Table S2** **Primer sequences**

| Gene | Primer sequence(5’-3’) |
| --- | --- |
| METTL3 | F: AGGCAGCTCATCTGTGTCCT  R: GCTTGGCGTGTGGTCTTT |
| PANC754 | F: TGGGCTTTGACAAACACTG  R: TCGGGAGGACCATCACTTA |
| PSPC1 | F: TGGAATTACAGGCAGATG  R: GTAGACGGACATTAAGGT |
| LGALS7 | F: GGGACGCCCAGTACCACCACT  R: TTCTGCTCAGAAGATCCTCACGGA |
| GAPDH | F: GGACCAATACGACCAAATCCG  R: AGCCACATCGCTCAGACAC |
| U6 | F: AGAGAAGATTAGCATGGCCCCTG  R: AGTGCAGGGTCCGAGGTATT |
| β-actin | F: ACTGGAACGGTGAAGGTGAC  R: AGAGAAGTGGGGTGGCTTTT |

**Table S3 meRIP-PCR primer sequences**

| Gene | Primer sequence(5’-3’) |
| --- | --- |
| PANC754 | F: GGCTAAAAATGAGGCAGA  R: TTGAAACCAAATGTTCCACC |

**Table S4** **siRNA and shRNA core sequences**

| Gene | shRNAcoresequence (5’-3’) |
| --- | --- |
| METTL3 | siMETTL3-1: GAGUGAUAUUUGUACAAUATT  siMETTL3-2: GGUUGCACGGUUCAAGCAATT |
| PANC754 | siPANC754-1:GGAAGCTTTGTCCAGTGATCG  siPANC754-2:GGAAATGCTTTCAGCTCTTT |
| PSPC1 | siPSPC1-1: GCTAATGAGGCAAGATCTAAT  siPSPC1-2: CCTAATAAGCGTCGTAGATAT |

**Table S5 The sense and antisense chain PCR primers for the PANC754 DNA template for in vitro transcriptionin RNA pull-down assay**

| PANC754 | | Primer sequence(5’-3’) |
| --- | --- | --- |
| Sense | F | taatacgactcactatagggACAATGAAAAACTAGACAGCCAATGGCTCA |
|  | R | AACTGGAACTGTTCTATTGGTATACTGGCA |
| Antisense | F | taatacgactcactatagggAACTGGAACTGTTCTATTGGTATACTGGCA |
|  | R | ACAATGAAAAACTAGACAGCCAATGGCTCA |

**Table S6 The proteins of solely binding to the sense strand of PANC754**

| **Accession** | **Description** | **Score Sequest HT: Sequest HT** | **# Peptides (by Search Engine): Sequest HT** | **Abundances (Normalized): F2: Sample, PANC754-sense** |
| --- | --- | --- | --- | --- |
| G3V4P8 | Glia maturation factor beta (Fragment) OS=Homo sapiens OX=9606 GN=GMFB PE=1 SV=1 | 1.66 | 1 | 1789343.75 |
| Q8WXF1 | Paraspeckle component 1 OS=Homo sapiens OX=9606 GN=PSPC1 PE=1 SV=1 | 6.71 | 2 | 1043424.688 |
| P11586 | C-1-tetrahydrofolate synthase, cytoplasmic OS=Homo sapiens OX=9606 GN=MTHFD1 PE=1 SV=3 | 8.65 | 3 | 927729.9844 |
| P31689 | DnaJ homolog subfamily A member 1 OS=Homo sapiens OX=9606 GN=DNAJA1 PE=1 SV=2 | 4.06 | 2 | 866243.1875 |
| P51610 | Host cell factor 1 OS=Homo sapiens OX=9606 GN=HCFC1 PE=1 SV=2 | 1.95 | 1 | 709945.6875 |
| A0A6I8PU89 | DnaJ homolog subfamily C member 7 OS=Homo sapiens OX=9606 GN=DNAJC7 PE=4 SV=1 | 3.7 | 1 | 612136.1875 |
| Q5T7C4 | High mobility group protein B1 OS=Homo sapiens OX=9606 GN=HMGB1 PE=1 SV=1 | 5.55 | 1 | 548293.8906 |
| Q14195 | Dihydropyrimidinase-related protein 3 OS=Homo sapiens OX=9606 GN=DPYSL3 PE=1 SV=1 | 8.62 | 4 | 444071.7188 |
| O75694 | Nuclear pore complex protein Nup155 OS=Homo sapiens OX=9606 GN=NUP155 PE=1 SV=1 | 4.01 | 1 | 414582.9063 |
| Q9Y295 | Developmentally-regulated GTP-binding protein 1 OS=Homo sapiens OX=9606 GN=DRG1 PE=1 SV=1 | 2.02 | 1 | 364842.3438 |
| O60841 | Eukaryotic translation initiation factor 5B OS=Homo sapiens OX=9606 GN=EIF5B PE=1 SV=4 | 0 | 1 | 323994.875 |
| Q9Y4L1 | Hypoxia up-regulated protein 1 OS=Homo sapiens OX=9606 GN=HYOU1 PE=1 SV=1 | 2.39 | 1 | 273684.375 |
| F6T1Q0 | 2',5'-phosphodiesterase 12 OS=Homo sapiens OX=9606 GN=PDE12 PE=1 SV=1 | 0 | 1 | 271060.7813 |
| Q8TC07 | TBC1 domain family member 15 OS=Homo sapiens OX=9606 GN=TBC1D15 PE=1 SV=2 | 1.79 | 1 | 247858.2031 |
| O43747 | AP-1 complex subunit gamma-1 OS=Homo sapiens OX=9606 GN=AP1G1 PE=1 SV=5 | 4.54 | 1 | 243162.8438 |
| O60763 | General vesicular transport factor p115 OS=Homo sapiens OX=9606 GN=USO1 PE=1 SV=2 | 1.85 | 1 | 240770.2188 |
| A0A0A0MSQ0 | Plastin-3 OS=Homo sapiens OX=9606 GN=PLS3 PE=1 SV=1 | 10.67 | 2 | 220509.1719 |
| Q9UBB4 | Ataxin-10 OS=Homo sapiens OX=9606 GN=ATXN10 PE=1 SV=1 | 0 | 1 | 216794.5938 |
| Q9Y5M8 | Signal recognition particle receptor subunit beta OS=Homo sapiens OX=9606 GN=SRPRB PE=1 SV=3 | 0 | 2 | 216366.0469 |
| E9PB61 | THO complex subunit 4 OS=Homo sapiens OX=9606 GN=ALYREF PE=1 SV=1 | 3.33 | 1 | 211860.1094 |
| H3BNW0 | THUMP domain-containing protein 1 OS=Homo sapiens OX=9606 GN=THUMPD1 PE=1 SV=1 | 1.92 | 1 | 211248.9688 |
| E9PK86 | Serpin H1 (Fragment) OS=Homo sapiens OX=9606 GN=SERPINH1 PE=1 SV=1 | 0 | 1 | 194685.1875 |
| P36952 | Serpin B5 OS=Homo sapiens OX=9606 GN=SERPINB5 PE=1 SV=2 | 1.75 | 1 | 190206.7813 |
| Q9H3K2 | Growth hormone-inducible transmembrane protein OS=Homo sapiens OX=9606 GN=GHITM PE=1 SV=2 | 1.69 | 1 | 183165.2813 |
| Q9NR30 | Nucleolar RNA helicase 2 OS=Homo sapiens OX=9606 GN=DDX21 PE=1 SV=5 | 0 | 1 | 181082.6406 |
| Q9Y2L1 | Exosome complex exonuclease RRP44 OS=Homo sapiens OX=9606 GN=DIS3 PE=1 SV=2 | 1.83 | 1 | 178564.7031 |
| F8VV59 | Nucleosome assembly protein 1-like 1 OS=Homo sapiens OX=9606 GN=NAP1L1 PE=1 SV=1 | 1.67 | 1 | 177784.7813 |
| P09543 | 2',3'-cyclic-nucleotide 3'-phosphodiesterase OS=Homo sapiens OX=9606 GN=CNP PE=1 SV=2 | 1.65 | 1 | 176717.2188 |
| A0A087X2I1 | 26S proteasome regulatory subunit 10B OS=Homo sapiens OX=9606 GN=PSMC6 PE=1 SV=1 | 0 | 2 | 171727.8438 |
| Q14CX7 | N-alpha-acetyltransferase 25, NatB auxiliary subunit OS=Homo sapiens OX=9606 GN=NAA25 PE=1 SV=1 | 0 | 1 | 170964.1875 |
| Q6NZI2 | Caveolae-associated protein 1 OS=Homo sapiens OX=9606 GN=CAVIN1 PE=1 SV=1 | 0 | 1 | 169306.7813 |
| Q9NP72 | Ras-related protein Rab-18 OS=Homo sapiens OX=9606 GN=RAB18 PE=1 SV=1 | 0 | 1 | 160013.2188 |
| O00303 | Eukaryotic translation initiation factor 3 subunit F OS=Homo sapiens OX=9606 GN=EIF3F PE=1 SV=1 | 0 | 2 | 157754.6563 |
| Q07065 | Cytoskeleton-associated protein 4 OS=Homo sapiens OX=9606 GN=CKAP4 PE=1 SV=2 | 0 | 1 | 140508.25 |
| P52306 | Rap1 GTPase-GDP dissociation stimulator 1 OS=Homo sapiens OX=9606 GN=RAP1GDS1 PE=1 SV=3 | 1.84 | 2 | 133783.2656 |
| Q9UL25 | Ras-related protein Rab-21 OS=Homo sapiens OX=9606 GN=RAB21 PE=1 SV=3 | 5.07 | 2 | 128996.75 |
| K7EN82 | Glycylpeptide N-tetradecanoyltransferase 1 (Fragment) OS=Homo sapiens OX=9606 GN=NMT1 PE=1 SV=1 | 0 | 1 | 127897.9531 |
| Q96HC4 | PDZ and LIM domain protein 5 OS=Homo sapiens OX=9606 GN=PDLIM5 PE=1 SV=5 | 0 | 1 | 123445.8516 |
| E7EX73 | Eukaryotic translation initiation factor 4 gamma 1 OS=Homo sapiens OX=9606 GN=EIF4G1 PE=1 SV=1 | 0 | 2 | 106171.6328 |
| H0YA24 | Pleiotropic regulator 1 (Fragment) OS=Homo sapiens OX=9606 GN=PLRG1 PE=1 SV=1 | 0 | 1 | 98842.61719 |
| Q9BRP1 | Programmed cell death protein 2-like OS=Homo sapiens OX=9606 GN=PDCD2L PE=1 SV=1 | 0 | 1 | 76211.96094 |
| Q96BW5 | Phosphotriesterase-related protein OS=Homo sapiens OX=9606 GN=PTER PE=1 SV=1 | 5.2 | 2 | 74247.6875 |
| Q9UKV8 | Protein argonaute-2 OS=Homo sapiens OX=9606 GN=AGO2 PE=1 SV=3 | 0 | 1 | 73652.39844 |
|  |  |  |  |  |
