# Supplementary material for: M6A-METTL3-dependent nuclear PANC754/PSPC1/H3K4me1 repression complex regulate immune evasive LGALS7 signal to enhance immunotherapy against colorectal cancer
Source: Cell Death Dis. 2025 Jul 9;16(1):506. doi: 10.1038/s41419-025-07820-9 (PMC12241656; doi:10.1038/s41419-025-07820-9)
Supplement: Supplementary file 3 — Supplemental Material file about WB [file 41419_2025_7820_MOESM3_ESM.pdf]

M[6]A-METTL3-dependent nuclear PANC754/PSPC1/H3K4me1 repression complex regulate immune evasive LGALS7 signal to enhance immunotherapy against colorectal cancer

Western blots

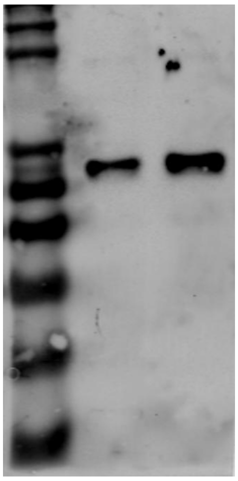

Uncropped E-cadherin WB

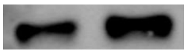

E-cadherin WB in Figure 1J

WB Figure S1. WB E-cadherin corresponded to Figure 1J

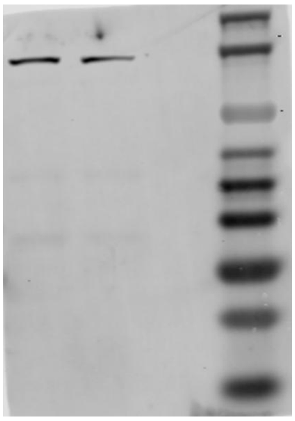

Uncropped MMP-9 WB

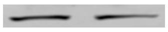

MMP9 WB in Figure 1J

WB Figure S2. WB MMP9 corresponded to Figure 1J

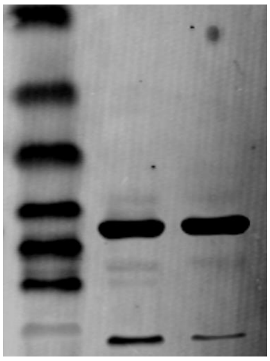
Uncropped GAPDH WB

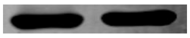
GAPDH WB in Figure 1J

WB Figure S3. WB GAPDH corresponded to Figure 1J

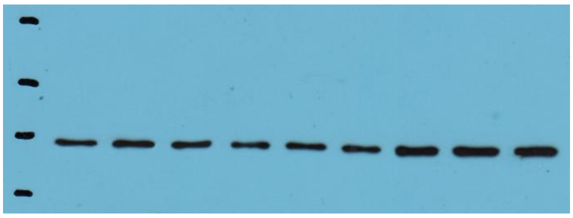
Uncropped E-cadherin WB

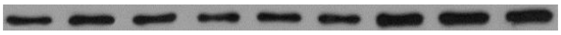
E-cadherin WB in Figure 2F

WB Figure S4. E-cadherin corresponded to Figure 2F

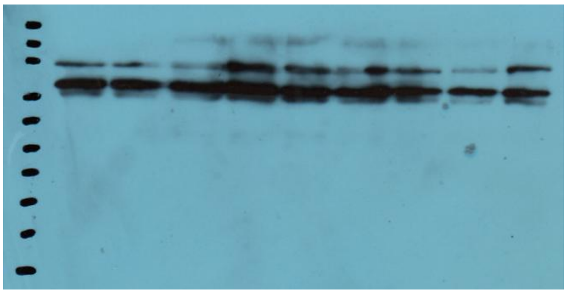
Uncropped MMP-9 WB

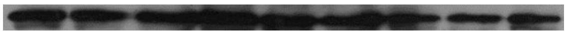
MMP-9 WB in Figure 2F

WB Figure S5. WB MMP-9 corresponded to Figure 2F

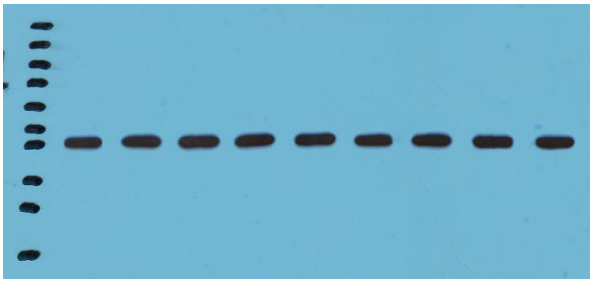
Uncropped GAPDH WB

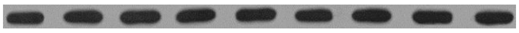
GAPDH WB in Figure 2F

WB Figure S6. WB GAPDH corresponded to Figure 2F

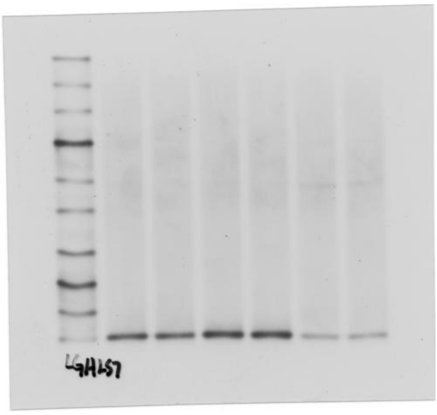

Uncropped LGALS7 WB

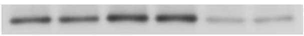

LGALS7 WB in Figure 5G

WB Figure S7. WB LGALS7 corresponded to Figure 5G

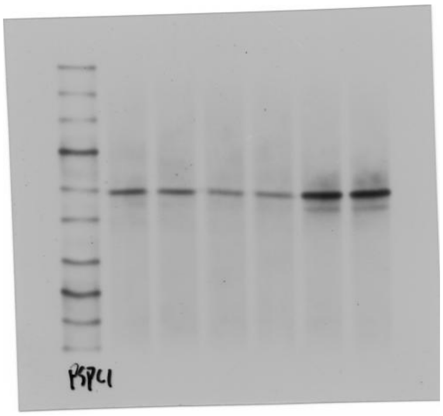

Uncropped PSPC1 WB

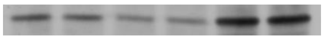

PSPC1 WB in Figure 5G

WB Figure S8. WB PSPC1 corresponded to Figure 5G

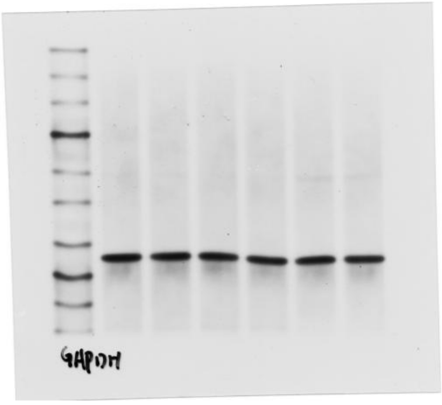

Uncropped GAPDH WB

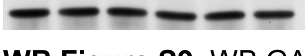

GAPDH WB in Figure 5G

WB Figure S9. WB GAPDH corresponded to Figure 5G

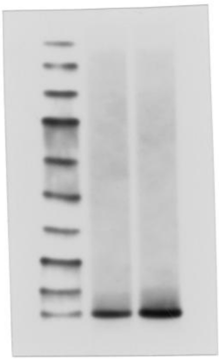

Uncropped H3K4me1 WB

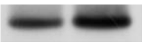

H3K4me1 WB in Figure 6B

WB Figure S10. WB H3K4me1 corresponded to Figure 6B

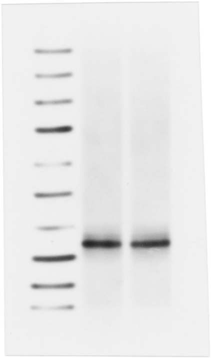

Uncropped PCNA WB

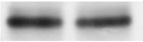

PCNA WB in Figure 6B

WB Figure S11. WB PCNA corresponded to Figure 6B

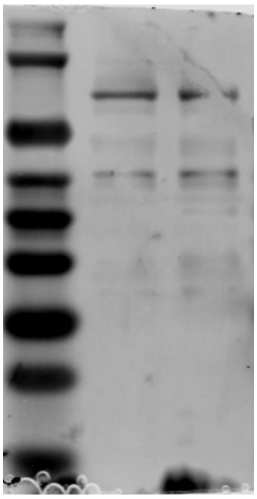

Uncropped N-Cadherin WB

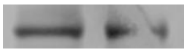

N-Cadherin WB in Figure S1-2D

WB Figure S12. WB N-Cadherin corresponded to Figure S1-2D

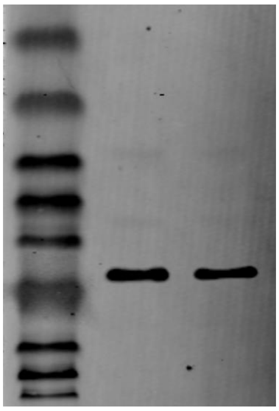
Uncropped Vimentin WB

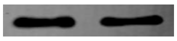
Vimentin WB in Figure S1-2D

WB Figure S13. WB Vimentin corresponded to Figure S1-2D

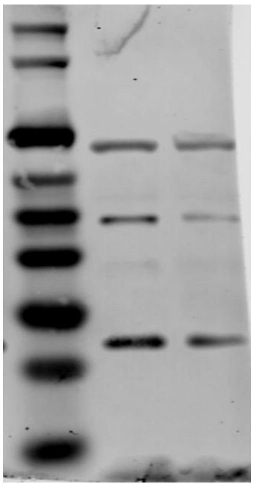
Uncropped SNAIL WB

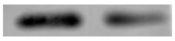
SNAIL WB in Figure S1-2D

WB Figure S14. WB SNAIL corresponded to Figure S1-2D

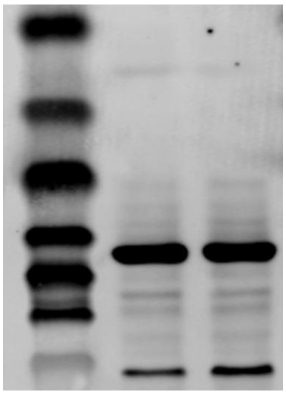
Uncropped GAPDH WB

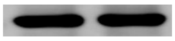
GAPDH WB in Figure S1-2D

WB Figure S15. WB GAPDH corresponded to Figure S1-2D

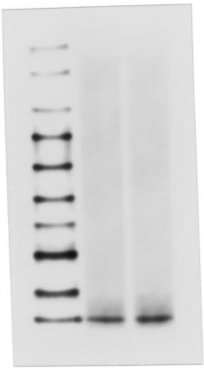
Uncropped H3K4ac WB

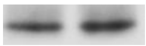
H3K4ac WB in Figure S6-3

WB Figure S16. WB H3K4ac corresponded to Figure S6-3

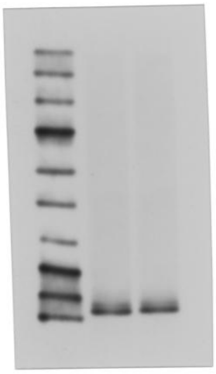
Uncropped Histone H3 WB

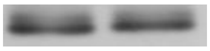
Histone H3 WB in Figure S6-3

WB Figure S17. WB Histone H3 corresponded to Figure S6-3
